# Development of a Bariatric Surgery Core Data Set for an International Registry

**DOI:** 10.1007/s11695-023-06545-y

**Published:** 2023-03-24

**Authors:** Karen D. Coulman, Katy Chalmers, Jane Blazeby, John Dixon, Lilian Kow, Ronald Liem, Dimitri J. Pournaras, Johan Ottosson, Richard Welbourn, Wendy Brown, Kerry Avery

**Affiliations:** 1 National Institute for Health Research Bristol Biomedical Research Centre, University of Bristol, Bristol, BS8 2BN UK; 2 Bristol Centre for Surgical Research, University of Bristol, Bristol, BS8 2PS UK; 3 Obesity and Bariatric Surgery Service, North Bristol NHS Trust, Bristol, BS10 5NB UK; 4 Iverson Health Innovation Research Institute, Swinburne University of Technology, Melbourne, 3122 Australia; 5 College of Medicine and Public Health, Flinders University, Adelaide, 5042 Australia; 6 Department of Surgery, Groene Hart Hospital, 2803 HH Gouda, The Netherlands; 7 School of Medical Sciences, Örebro University, 701 82 Örebro, Sweden; 8 Department of Upper GI and Bariatric Surgery, Somerset NHS Foundation Trust, Taunton, TA1 5DA UK; 9 Department of Surgery, Monash University, Melbourne, 3800 Australia

**Keywords:** Bariatric surgery, Core outcomes, Clinical registries

## Abstract

**Purpose:**

Bariatric and metabolic surgery is an effective treatment for severe and complex obesity; however, robust long-term data comparing operations is lacking. Clinical registries complement clinical trials in contributing to this evidence base. Agreement on standard data for bariatric registries is needed to facilitate comparisons. This study developed a Core Registry Set (CRS) — core data to include in bariatric surgery registries globally.

**Materials and Methods:**

Relevant items were identified from a bariatric surgery research core outcome set, a registry data dictionary project, systematic literature searches, and a patient advisory group. This comprehensive list informed a questionnaire for a two-round Delphi survey with international health professionals. Participants rated each item’s importance and received anonymized feedback in round 2. Using pre-defined criteria, items were then categorized for voting at a consensus meeting to agree the CRS.

**Results:**

Items identified from all sources were grouped into 97 questionnaire items. Professionals (*n* = 272) from 56 countries participated in the round 1 survey of which 45% responded to round 2. Twenty-four professionals from 13 countries participated in the consensus meeting. Twelve items were voted into the CRS including demographic and bariatric procedure information, effectiveness, and safety outcomes.

**Conclusion:**

This CRS is the first step towards unifying bariatric surgery registries internationally. We recommend the CRS is included as a minimum dataset in all bariatric registries worldwide. Adoption of the CRS will enable meaningful international comparisons of bariatric operations. Future work will agree definitions and measures for the CRS including incorporating quality-of-life measures defined in a parallel project.

**Graphical Abstract:**

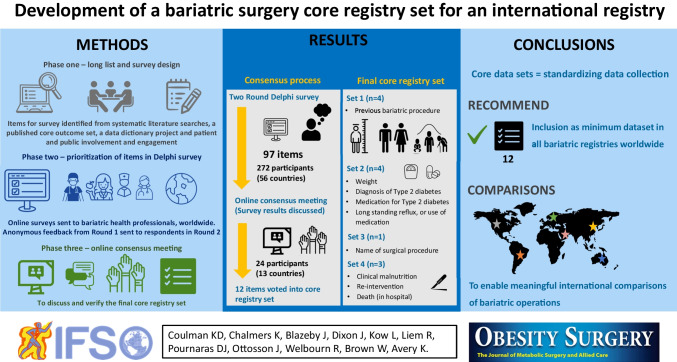

**Supplementary Information:**

The online version contains supplementary material available at 10.1007/s11695-023-06545-y.

## Introduction

Obesity rates have tripled internationally since 1975 [1]. In the USA, Australia, and the UK, adult obesity rates are 42%, 31%, and 29%, respectively, with the highest rates found in the Pacific islands and the Middle East [2–5]. Obesity is associated with an increased risk of type 2 diabetes, cardiovascular disease, different cancers, and premature death [6, 7]. Within this population, people with severe and complex obesity (BMI ≥ 40 kg/m^2^, or 35–40 kg/m^2^ with another significant health problem that could be improved by weight loss) suffer the greatest health burdens and are at the highest risk of premature death [8, 9].

Bariatric surgery, combined with behavior change and dietary management, is currently the most durably effective treatment for people with severe and complex obesity [9–11]. There are several different types of surgery. Recent international data indicate that sleeve gastrectomy (SG, 67%) is the most common operation, followed by the Roux-en-Y gastric bypass (RYGB, 24%), with adjustable gastric band (AGB, 0.8%) decreasing in recent years, and the one-anastomosis gastric bypass (OAGB, 4.5%) gaining popularity [12]. Each of these procedures works slightly differently; mechanisms include alteration of gut hormone levels that lead to reduction in hunger, improvement in satiety, and shifts in food preferences, associated with changes in bile acids and vagal signaling [13].

There is, however, a paucity of data from large, well designed and conducted randomized controlled trials (RCTs) or matched cohort studies comparing the effectiveness and safety of the different types of operations. Published studies also suffer from problems with heterogeneity of outcome selection, measurement, and reporting. This makes comparison of data from different studies challenging [10, 14, 15]. To help address these issues, a core outcome set to be used in effectiveness trials of bariatric surgery was previously developed [16]. Following on from this work, the international SQOT (standardizing quality of life measures in obesity treatment) initiative is undertaking research to determine the most appropriate patient-reported outcome measures for quality of life (QOL) in obesity treatment – one of the core outcomes identified in the bariatric surgery core outcome set [17–19].

Although double-blinded RCTs are considered the gold standard study design to compare interventions, they can be expensive and challenging to conduct, especially in surgery [20, 21]. For example, blinding of surgeons and subjects to treatment type may not be possible, whilst randomization may not be acceptable to participants. Well-collected prospective data from clinical registries provide important ‘real-world’ data to complement RCTs. Clinical registries may be used to examine disease epidemiology, treatment effectiveness and the quality of patient care. Data recorded in registries may include demographic, diagnostic, prognostic or technical variables, as well as clinically important outcomes, including both effectiveness outcomes and complication rates for surgical interventions [22]. Additionally, RCTs can be nested within existing registries which can improve trial recruitment [21].

There are at least 18 national bariatric surgery registries in existence; however, not all have been developed with key quality indicators defined at the outset [22–24]. As with published research studies in this area, variables collected in different registries, including how they are defined and measured, vary considerably between countries, making cross-country comparisons difficult [22–24]. Akpinar et al. found that only 10% of variables collected in bariatric surgery registries had perfect agreement across registries, highlighting a clear need for a standardized minimum set of data items to be agreed and implemented across registries [24]. The International Federation for the Surgery of Obesity and Metabolic Disorders (IFSO) has established a global registry project to allow for comparisons of obesity and bariatric surgery on an international level [22, 23]. The registry aims to ‘work towards providing the most credible and transparent information available on bariatric and metabolic surgery’ [12]. At present, however, reporting is limited to demographic and procedural data and there are important differences in how the data elements are collected and defined [12, 25]. These differences make meaningful comparisons between different populations challenging. Bariatric surgery registries are not alone in this challenge; however, core data sets have been developed for registries in other clinical areas to improve comparability of data [26–29].

An agreed standardized set of data items (core set) that should be measured and reported by all bariatric surgery registries in a consistent fashion is needed to enable more comprehensive comparison of bariatric surgery registry data on an international level [22, 23, 25]. A core set does not imply that registries should restrict what they measure to only the items in the core set. Rather, there is an expectation that the core items will be collected and reported making it easier for data from different bariatric surgery registries to be combined and compared, while other items are measured as well [30]. The aim of this project was to develop a core set of data items or Core Registry Set (CRS) for use in bariatric surgery registries.

## Materials and Methods

The CRS was developed according to principles outlined in the Core Outcome Measures for Effectiveness Trials (COMET) Handbook and Core Outcome Set-Standards for Development (COS-STAD) guidelines [31, 32]. Whilst designed to identify core ‘outcome’ sets, the methods have been used for other types of data — e.g., core ‘information’ sets as they provide a structure and process for comprehensively examining all candidate items and reducing them down using scientific consensus methods. This methodology has previously been used to delineate core sets of data for disease registries in other clinical areas [26, 27]. The study comprised three phases: (1) development of a comprehensive (long) list of items relevant to the monitoring and evaluation of bariatric surgery to be used within a Delphi questionnaire, 2) prioritization of the long list of items in a Delphi survey with key international multi-disciplinary stakeholders, 3) further prioritization and agreement of the final core set with stakeholders at a consensus meeting. The full study protocol is included in Supplementary File 1.

The study team did not intend to reach consensus on core patient-reported outcomes to be included in the international registry, due to parallel ongoing work by the SQOT initiative to achieve global consensus on patient-reported outcome measures for obesity treatments in which several of this study’s authors are involved [18]. The work being undertaken by the SQOT initiative includes strong participation from people living with obesity. Thus, the focus of this project was on non-patient-reported outcomes.

### Phase 1: Development of the Long List of Items and Questionnaire for a Delphi Survey

A comprehensive long list of potentially relevant items to include in the CRS was generated from the following data sources:

(i) Items identified during the development of an existing COS for bariatric surgery effectiveness trials using established consensus methodology in the BARIACT study [16]. Items included in the BARIACT study were identified from three systematic reviews [14, 15, 33] and qualitative interviews with patients that had undergone bariatric surgery [34], the methods for which have previously been reported in full [16, 35].
(ii) Items identified from a Dutch data dictionary project, which collated items from 11 existing national bariatric surgery registries from the following countries: Australia and New Zealand, Austria, Brazil, Kuwait, Mexico, the Netherlands, Norway and Sweden, Russia, Turkey, UK, and USA [24].
(iii) Items identified from annual systematic searches of bariatric surgery effectiveness trials covering the years 2013–2020 (unpublished data) to inform the By-Band-Sleeve study [36].

Items identified from each data source were combined into a single long list, and any duplicates removed or overlapping items combined by the study team, which included clinical scientists (e.g., methodologists) and health professionals (KCo, KCh, JB, JO, RW, KA). Items were mapped onto broader domains as previously described [16]. The study team then grouped the long list of domains and items into three lists representing the different phases of data collection within bariatric surgery registries: 1. Baseline data (e.g. items that will only be measured at baseline, such as demographics); 2. Effectiveness outcomes (measured at baseline and follow-up); 3. (a) Surgical procedure information (measured only once peri-operatively) and (b) surgical complications (measured during and/or after surgery). The patient and public involvement (PPI) group reviewed the long list and were able to suggest additional items they felt were important but not already included.

These three lists were used to draft a questionnaire structured into four main sections: core set 1–baseline only information; core set 2: effectiveness outcomes; core set 3a: surgical procedure information; core set 3b: potential complications and side effects of surgery. Each item from the lists formed an individual item within the questionnaire. The broader domains formed categories of items within each section. Each item was accompanied by a nine-point Likert scale for rating the importance of including the item in the final core sets, labeled 1 to 3 ‘limited importance’, 4 to 6 ‘important but not critical’, and 7 to 9 ‘critical importance’, based on the grading of recommendations assessment, development and evaluation (GRADE) guidelines [37]. Additional free text items were included to enable stakeholders to propose new items. Survey questionnaires were reviewed by the study management group (SMG) and PPI group to ensure clarity and acceptability. In the first survey round, additional items were included to explore stakeholder views on follow-up timepoints for outcome data collection for the effectiveness core set and the surgical complications core set, although this exploratory sub-study was not designed to achieve consensus on this area (findings to be reported separately).

### Phase 2: Prioritization of Items in an International Delphi Survey

To achieve consensus on the items to be included in the CRS a two-round Delphi survey was undertaken followed by a virtual consensus meeting (phase 3). The Delphi process was used to enable a diverse group of participants from a wide geographical area to participate, while preserving anonymity so as to prevent results from being strongly influenced by the views of dominating individuals [38]. Multidisciplinary health professionals involved in the care of bariatric surgery patients who were members of IFSO or one of IFSO’s member societies were invited to take part in the survey.

Stakeholders were invited to participate in the Delphi survey which included two sequential survey rounds, administered online using REDCap electronic data capture tools hosted at the University of Bristol [39, 40] in accordance with CHERRIES guidelines for electronic surveys [41]. Those who completed the round 1 questionnaire were eligible to complete round 2. Participants were asked to rate the importance of each questionnaire item on a nine-point scale ranging from one (limited importance) to nine (critical importance). Proposed new items recommended by two or more participants in round 1 were considered for inclusion in round 2 by the study team [42].

The round 2 questionnaire was identical to round 1 but also included personalized feedback from round 1, and any additional items proposed in round 1. Participants received their own individual round 1 scores for each item, the median scores of their peer group (groups included ‘Surgeon’, ‘Physician’, ‘Specialist Nurse’, ‘Dietitian’, ‘Psychologist’, ‘Other professional’), of all other health professionals (excluding their peer group), and of the whole group. Participants were asked to re-rate the items on the questionnaire, considering the round 1 feedback.

#### Survey Participants and Sampling

To ensure the resulting CRS was developed with international input from a range of multidisciplinary health professionals involved in bariatric surgery care, international health professionals (including surgeons, and integrated health professionals such as specialist nurses, dietitians, psychologists, and physicians) were invited through the IFSO membership. As explained above, this project did not focus on patient-reported outcomes, and thus, patients were not included as participants within the consensus process; however, an international PPI group of people who had undergone bariatric surgery advised on the project (see the “Patient and Public Involvement” section).

An email invitation to participate in the Delphi survey was sent to all IFSO members by the IFSO president. Presidents of the 66 official member societies of IFSO were also asked to send the invitation to their members. There is no agreed methodology for determining the sample size required for consensus processes to develop a core set. Sample size is dependent on the scope of a core set and decisions on the stakeholder groups to be involved, as well as practical feasibility considerations [31, 38]. The UK-based BARIACT project, which developed a COS for bariatric surgery effectiveness trials, included 168 health professionals in the Delphi survey and 33 participants in the professional consensus meeting [16]. This project was registered on the COMET (Core Outcome Measures in Effectiveness Trials) database [43].

The results of the round 2 survey were classified into three groups for presentation and review at the consensus meeting (see the “Statistical Analyses” section). This included (1) items that were rated 7–9 by ≥ 95% of participants AND 1–3 by < 15% of participants, (2) items that were rated 7–9 by 70–95% of participants AND 1–3 by < 15% of participants, (3) items that were rated 7–9 by < 70% of participants (Table 1). The SMG and PPI group separately reviewed the results of the Delphi survey in advance of the consensus meeting and were able to highlight any items in this latter group (group 3) that they wanted to ‘save’ for potential inclusion including in the CRS. This information was provided to consensus meeting participants in advance of the meeting.

**Table 1:** Classification of items from Delphi survey results, including action at consensus meeting

Group	Classification of items	Consensus meeting action
1	Items rated 7–9 by ≥ 95% of all participants AND 1–3 by < 15% of all participants Met consensus threshold to be automatically included in Core Registry Set (CRS)	Ratify ‘IN’: Only discussed and voted on if anyone raised objections to them being included in the CRS (otherwise they were automatically included in the CRS)
2	Items rated 7–9 by 70–95% of all participants AND 1–3 by < 15% of all participants Met consensus threshold to be automatically included for discussion in the consensus meeting	Discussed in small groups whereby each group proposed their top 3 items to be included in the CRS. All items proposed were then discussed and voted on by the whole group
3	Items rated 7–9 by < 70% of all participants Did not meet consensus threshold to be automatically included in the CRS or discussed in the consensus meeting	Ratify ‘OUT’: Only discussed and voted on if anyone raised objections to them NOT being included in the CRS (otherwise they were NOT included in the CRS)

### Phase 3: Consensus Meeting

Participants in round 2 of the Delphi survey were asked to indicate their interest in taking part in an online consensus meeting to finalize the CRS. Those who indicated their interest on the round 2 survey were invited to take part in the meeting. One week prior to the meeting, participants were provided with a pre-meeting information pack (Supplementary File 2) which included an agenda, a description of the project, the main objective of the consensus meeting, and the results of the round 2 survey as classified into the three groups described above. This included any items in group 3 that the SMG or PPI group had highlighted they would like to save for possible inclusion in the CRS. Consensus meeting participants were given the opportunity to contact the meeting organizers prior to the meeting with any of these items that they wished to ‘save’ for discussion at the consensus meeting (i.e., that they objected to being excluded from the core set).

The consensus meeting was held virtually over Zoom on November 20th, 2021, and was chaired by an independent expert in core set development methodology with previous experience of running consensus meetings. The meeting began with an introduction including a description of the work undertaken to date, and how the meeting would run. Discussion and voting on items then took place for each of the four main sections of the survey (see Phase 1). For each section or core set, we began with a whole group discussion and voting (where needed) on items in group 1. This was followed by small group discussion in breakout groups where each small group selected their top 3 items from group 2. Each small group’s top 3 was fed back to the wider group. All items fed back were then discussed and voted on by the full group. For items to be voted on, participants were asked to vote ‘Yes’ (this item should be included in the CRS) or ‘No’ (this item should not be included in the CRS’. Voting was undertaken anonymously using the Polls function in Zoom. Once all participants had cast their votes, the results were presented to the group for immediate feedback. At the end of the meeting, all items voted in (see the “Statistical Analyses” section) were presented to meeting participants for discussion and finalization of the CRS.

### Statistical Analyses

Descriptive statistics were used to summarize the results of rounds 1 and 2 of the Delphi survey. The median score for each item in round 1 was calculated for each professional sub-group and presented as feedback for the Round 2 questionnaire. After Round 2, the percentage of participants rating each outcome 7–9 (critical importance) was calculated for ‘All participants’, and the sub-groups ‘Surgeons’ and ‘Other professionals’ (excluding surgeons). Items that were rated 7–9 by ≥ 70% of ‘All Participants’ met the consensus threshold to be automatically included for discussion in the consensus meeting. Items were classified into three groups as per Table 1 to aid with the running of the consensus meeting. Items voted on at the consensus meeting were retained for each core set if ≥ 70% of meeting participants voted ‘Yes’ to include the item in the core set. Consensus limits were selected based on previous studies using consensus methods to develop core outcome sets [44, 45]. All statistical analyses were undertaken using STATA 15 statistical software [46].

### Patient and Public Involvement

A separate international Patient and Public Involvement (PPI) group consisting of seven people living with obesity who had undergone bariatric surgery was formed to provide guidance on the different phases of the project. This group met separately to the professional SMG to ensure patient representatives were adequately able to express their views. Researchers met with the PPI group prior to the consensus process to review the draft questionnaire for the Delphi survey. The group were asked to suggest any important items not already included within the draft questionnaire and provide feedback on the clarity and acceptability of the questionnaire. Researchers met with the PPI group again after the Delphi survey and prior to the consensus meeting. The researchers reviewed the results of the Delphi survey with the PPI group and asked participants to highlight any items they felt were important that had not been ranked highly within the Delphi survey. Further details are provided throughout the Method and Results where relevant.

### Embedded methodological study

As part of an embedded methodological study to explore optimal methods for providing feedback to encourage prioritization between Delphi survey rounds, participants were randomized to receive one of two versions of the Round 2 questionnaire (basic or extended feedback) (Supplementary File 3). Participants randomized to ‘Enhanced feedback’ received the personalized feedback as described above plus feedback on the top five highly rated items from round 1 in each section of the questionnaire. Results will be reported separately.

## Results

**Fig. 1:**
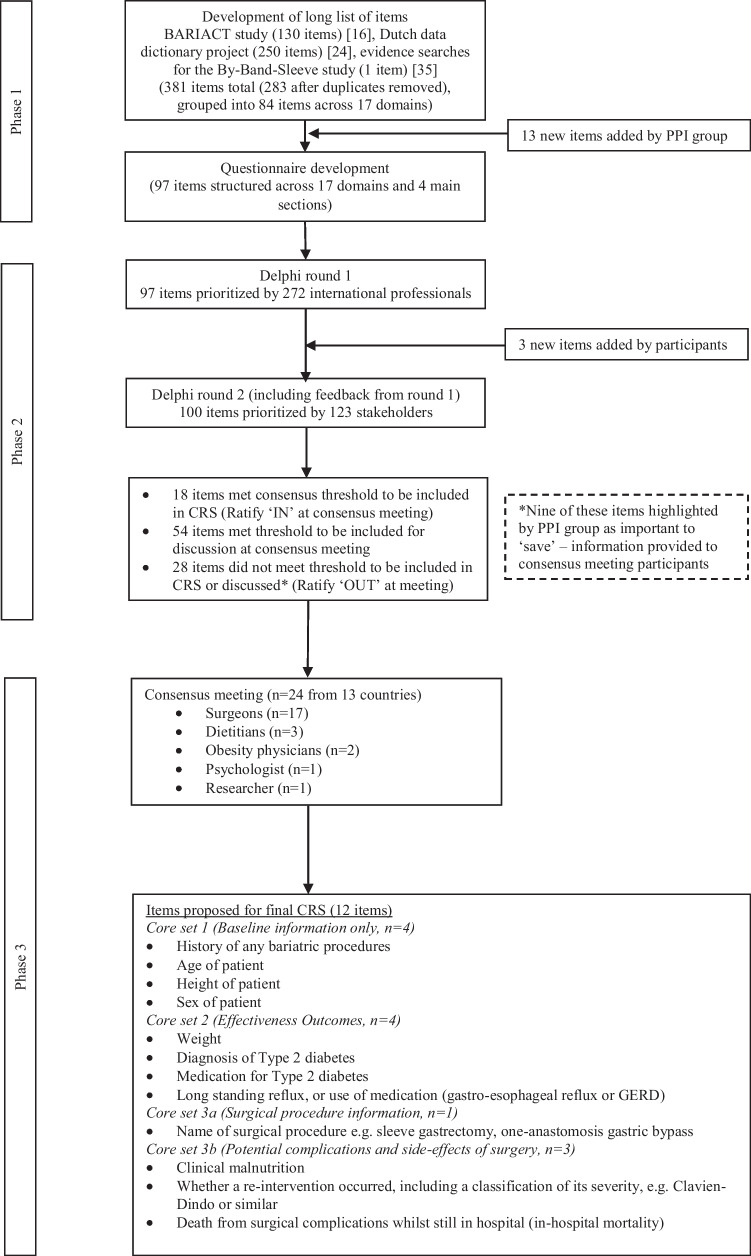
Summary of three study phases for the development of a Core Registry Set (CRS) for bariatric surgery

An overview of the main results for each phase of the study is presented in Fig. 1.

### Phase 1: Long List and Survey Development

The 130 items from the survey used in the BARIACT study were combined with 250 items identified in the Dutch data dictionary project [24]. One additional item (beta cell function) that was not included in the other two sources was added from a review of the literature associated with the By-Band-Sleeve study [36]. After removal of duplicates, 283 items remained which were collapsed into 84 broader items and categorized within 17 domains across the three lists in discussion with the SMG. From discussion with the PPI group, an additional 13 items were added to the long list (Table 2). The final round 1 questionnaire included 97 items within 17 domains, structured across four main sections (Table 3). An additional question was added at the end of each section where participants could suggest new items for a total of 101 items in the questionnaire. The full rounds 1 and 2 questionnaires are available as Supplementary File 3.

**Table 2:** Additional items added to long list by Patient and Public Involvement group

Core Set 1: Baseline only information (*n* = 1) • Time period over which pre-surgery weight loss occurred
Core Set 2: Effectiveness outcomes (*n* = 7) • Ability to fall asleep at night or quality of sleep (sleep disorders other than sleep apnea) • Long standing fluid retention (lymphedema) • Abnormal accumulation of fat in legs/arms (lipedema) • Feelings towards one’s body shape or appearance (body dysmorphia/dysmorphic disorder) • Addictive behaviors, e.g., alcohol, gambling, illicit drugs • Anger management problems • Changes in family and relationship
Set 3b: Potential complications and side-effects of surgery (*n* = 5) • Problems with teeth • Hair loss • Problems with kidney stones • Leg cramps • Problems with immune system, e.g., recurrent infections Additionally, the wording of one item in set 3b was elaborated: Skin problems or irritations, e.g., rashes, sores, loose skin, or ulcers or exacerbation of existing skin problems

**Table 3:** Round 1 Delphi survey questionnaire structure and headings (n = 97)

**Core Set 1: Baseline only information (*n* = 15)** • Administrative information (*n* = 3) • Patient demographics (*n* = 5) • Clinical history (*n* = 7)
**Core Set 2: Effectiveness outcomes (*n* = 33)** • Obesity-related disease (*n* = 19) • Mental health status assessed by a health professional (*n* = 6) • Anthropometric (body measurement) data (*n* = 2) • Lifestyle data (*n* = 4) • Other outcomes (*n* = 2)
**Set 3a: Surgical procedure information (surgeons only) (*n* = 17)** • General surgical information (*n* = 5) • Stapling/suturing procedures (*n* = 8) • Device procedures (*n* = 4)
**Set 3b: Potential complications and side-effects of surgery (*n* = 32)** • Death (*n* = 3) • Technical complications of stapling/suturing procedures (*n* = 2) • Technical complications related to operations using devices (*n* = 2) • General complications of surgery (early post-operative period) (*n* = 9) • Side effects of surgery (longer-term) (*n* = 13) • Nutritional outcomes (*n* = 3)

### Phase 2: Delphi Survey

A total of 272 professionals, from 56 countries, took part in the round 1 survey (Table 4). Of these, 123 responded to round 2 (45.2%). Seventy and ninety-one percent of participants answered all survey items in rounds 1 and 2, respectively. Three additional items were added to round 2 as proposed by participants in round 1, including ‘Medication history’ (core set 1), history of any previous abdominal surgery (other than bariatric surgery)’ (core set 1), and ‘Physical activity levels’ (core set 2). The top five items in each core set after rounds 1 and 2 are presented in Table 5, with full results for all items in the Supplementary File 4. After the Delphi survey, 18 items met the consensus threshold to be included in the CRS (group 1 — ‘Ratify IN’ at consensus meeting), 54 met the threshold to be included for discussion in the consensus meeting (group 2), and 28 did not meet threshold to be included in the CRS or discussed in the consensus meeting (group 3 — ratify ‘OUT’ at consensus meeting) (Fig. 1). The PPI group highlighted nine of the 28 items in group 3 to ‘save’ for the consensus meeting: ability of patient to purchase/afford supplements for life, post-surgery; ethnicity of the patient; changes in family and relationship; long standing fluid retention (lymphedema); abnormal accumulation of fat in legs/arms (lipedema); problems with bowel movements/flatulence; problems with teeth; problems with kidney stones; skin problems or irritations, e.g., rashes, sores, loose skin or ulcers or exacerbation of existing skin problems*.* These items were highlighted in the pre-meeting information pack which consensus meeting attendees received.

**Table 4:** Delphi survey respondents

Specialty	Round 1^1^		Round 2^2^	
	Total number of responses	Stakeholder representation (%)	Total number of responses	Stakeholder representation (%)
Surgeon	156	57.3	68	55.3
Bariatric physician	14	5.1	5	4.1
Specialist nurse	16	5.9	7	5.7
Dietitian	58	21.3	28	22.8
Psychologist	11	4.0	7	5.7
Other	16	5.9	8	6.5
Total	**272**		**123 (45.2%)**	

^1^Sent to International Federation for the Surgery of Obesity and Metabolic Disorders (IFSO) membership, ^2^Sent to round 1 respondents

**Table 5:** Top 5 items* for each core set after rounds 1 and 2 of the Delphi survey

Round 1	Round 2
Core set 1 — baseline information only	
1. History of previous types of bariatric surgery	1. History of any previous bariatric surgery
2. Duration of type 2 diabetes	2. Height of the patient
3. Other medical conditions not directly related to obesity	3. Other medical conditions not directly related to obesity e.g., type 1 diabetes, dementia
4. Age of the patient	4. Age of patient
5. Height of the patient	5. Medication history
Core set 2 — effectiveness outcomes	
1. Diagnosis of Type 2 diabetes	1. Diagnosis of type 2 diabetes
2. Weight	2. Weight
3. Medication for type 2 diabetes	3. Addictive behaviors, e.g., alcohol, gambling, illicit drugs
4. Problems with breathing during sleep (obstructive sleep apnea)	4. Medication for type 2 diabetes
5. Long standing acid reflux, or use of medication (gastro-esophageal reflux or GERD)	5. Problems with breathing during sleep (obstructive sleep apnea)
Core set 3a — surgical procedure information	
1. Name of surgical procedure, e.g., sleeve gastrectomy, one-anastomosis gastric bypass	1. Measurements of limb length (not for sleeve gastrectomy)
2. Surgical approach to gain access e.g., laparoscopic, open or endoscopic	2. Name of surgical procedure, e.g., sleeve gastrectomy, one-anastomosis gastric bypass
3. Closure of potential internal hernia defects undertaken (not for sleeve gastrectomy)	3. Closure of hernia defects undertaken (not for sleeve gastrectomy)
4. Measurements of limb length (not for sleeve gastrectomy)	4. Surgical approach to gain access, e.g., laparoscopic, open or endoscopic
5. Hiatus hernia repair undertaken	5. Duration of balloon implantation (when removed)
Core set 3b — potential complications and side-effects of surgery	
1. Death from surgical complications whilst still in hospital (in-hospital mortality)	1. Clinical malnutrition
2. Obstruction including ileus and/or hernia (stapling/suturing procedures only)	2. Whether a re-intervention occurred, including a classification of its severity, e.g., Clavien-Dindo or similar
3. Complications that may occur shortly after the operation when the patient is still in hospital (device operations only)	3. Bleeding inside the body (intra-abdominal or endoluminal)
4. Death after discharge from hospital (post-discharge mortality)	4. Problems with the heart, vessels, or blood clots (cardiovascular problems or venous thromboembolism)
5. Cause of death	5. Problems with anastomotic/staple line/suture line including subsequent infections

^*^As ranked by percentage of participants rating items 7–9 in Delphi survey

### Phase 3: Consensus Meeting

Of the 123 participants in the round 2 survey, 30 indicated their interest in attending the consensus meeting, of which 24 attended on the day (19.5%). Professionals from 13 countries (Argentina, Australia, Austria, Brazil, China, Egypt, India, Mexico, Netherlands, Norway, Sweden, UK, USA) were represented and included surgeons (*n* = 17), dietitians (*n* = 3), obesity physicians (*n* = 2), a psychologist (*n* = 1), and a researcher (*n* = 1). Six of the participants were SMG members.

Results of the voting for each item are presented in Supplementary File 4. The final 12 items voted into the CRS at the consensus meeting are presented in Fig. 1 and Table 6. The following key points were noted during the meeting, with agreement that these points would require further consideration: (1) consider combining ‘diagnosis of type 2 diabetes’ and ‘medication for type 2 diabetes’ items into a single item (core set 2), (2) consider combining complications items (core set 3b), (3) a separate discussion to be held on inclusion of cardiovascular risk/medications, (4) consideration to reviewing the core sets in the context of the SQOT study findings with regards to including the core measurement of psychological aspects within the CRS.

**Table 6:** Final items proposed for the bariatric surgery Core Registry Set

**Core set 1: Baseline information only (4 items)**
History of any bariatric procedures
Age of patient
Height of patient
Sex of patient
**Core set 2: Effectiveness outcomes (4 items)**
Weight
Diagnosis of type 2 diabetes
Medication for type 2 diabetes
Long standing reflux, or use of medication (gastro-esophageal reflux or GERD)
**Core set 3a: Surgical procedure information (1 item)**
Name of surgical procedure e.g. sleeve gastrectomy, one-anastomosis gastric bypass
**Core set 3b: Potential complications and side-effects of surgery (3 items)**
Clinical malnutrition
Whether a re-intervention occurred, including a classification of its severity, e.g., Clavien-Dindo or similar
Death from surgical complications whilst still in hospital (in-hospital mortality)

## Discussion

This study has developed an international CRS of items for bariatric surgery registries. This was informed from a comprehensive investigation into potential items, and consensus methodology with health professionals in 56 countries to prioritize the key items. The final 12 items in the CRS include baseline demographic and clinical information, clinical effectiveness, and safety outcomes. Findings will be amalgamated with an international project developing core QOL measures for obesity treatments [18]. The consensus meeting highlighted areas requiring further discussion including further grouping of some items, and possible inclusion of ‘cardiovascular risk’ and psychological outcomes which will be considered in future work.

Clinical registries have been demonstrated to improve patient safety, service delivery systems as well as reduce costs for payers [47–52]. The ability to compare data collected by registries offers the opportunity to understand national and international trends as well as potentially benchmark performance, providing the opportunity to learn from both positive and negative variance of practice. Strengthening registries with core data sets can help to harness their power in evaluating the comparative effectiveness of clinical treatments such as surgery [53]. Embedding core sets within registries, however, can present unique challenges. Registries may collect a wider range of data than RCTs making it difficult to limit the core set to a feasible number of items [26]. There may be national and institutional barriers to incorporating particular data items within registries, creating challenges for collecting standardized data on an international level [54]. Mindful of these challenges, participants in this study were able to prioritize 12 key items to include in the CRS. These potential challenges will continue to be considered in further work to define and select measures for items in the CRS.

Previous work has been undertaken to amalgamate national bariatric surgery registry data from the Netherlands, Sweden and Norway to compare outcomes of bariatric surgery [55, 56]. This showed considerable national variation in rates of some types of post-operative complications, re-interventions, re-admissions and length of stay, providing opportunity for improvement [55, 56]. These comparisons were possible due to uniform data elements and definitions across the three national registries. Whilst there are important data housed within all 18 national bariatric surgery registries, the ability to compare these data is currently limited by inconsistencies in the data items collected. Even when the names of data items collected are the same, the definitions often differ, making meaningful and valid comparisons challenging.

This study has identified 12 core items to include in bariatric registries, compared with nine core outcomes included within a COS for bariatric surgery effectiveness trials [16]. Five of the 12 items in the CRS (core sets 1 and 3a) represent ‘registry-specific’ items — demographic, clinical background, and procedure information that would be measured only once. The other seven items (core sets 2 and 3b) include clinical outcomes and adverse events to evaluate the effectiveness and safety of surgery which could also be included within a research COS. ‘Overall QOL’ was included within the COS, however, was purposefully not included within this study due to parallel work to define QOL measurement which will be amalgamated with this registry project [18]. ‘Cardiovascular risk’ was included within the COS but did not reach consensus to be included in the CRS. It was, however, highlighted at the consensus meeting as needing further discussion including the elements needed to ascertain cardiovascular risk such as type 2 diabetes (which is included in the CRS). Other items across the COS and CRS are related but worded differently such as ‘Diabetes status’ in the COS and ‘Diagnosis of Type 2 diabetes’ and ‘Medication for Type 2 diabetes’ in the CRS; ‘micronutrient status’ in the COS and ‘clinical malnutrition’ in the CRS; ‘Dysphagia/regurgitation’ in the COS and ‘Long standing reflux, or use of medication (gastro-esophageal reflux or GERD)’ in the CRS. Work is now needed to establish consistent wording of items and definitions across both the CRS and COS to allow for comparative effectiveness data from both sources to be combined. Another recent initiative is the Gastro-intestinal Coordinated Registry Network established in the USA to define a minimum core data structure for the collection of ‘real world’ data for obesity endoscopic procedures [57]. There is the potential for data from the CRS and COS to link in with this dataset. Core sets should be kept under review and revised where appropriate, for example, if registries are consistently measuring an item that is not in the core set, a revision or update might be indicated [31]. The American Society of Metabolic and Bariatric Surgery (ASMBS) together with IFSO have recently published a position statement with updated indications for metabolic and bariatric surgery [58]. Changes in clinical practice which may arise from this position statement may influence what items bariatric surgery registries collect in the years to come. Other novel concepts in the field of bariatric surgery have emerged in recent years, including textbook outcomes, global outcome benchmarks, and risk prediction models [59–62]. The CRS will be kept under review to incorporate important global changes in bariatric surgery clinical practice.

A major strength of our study is the use of rigorous established methods for core set development with participation from international stakeholders across 56 countries. A range of multidisciplinary health professionals involved in bariatric care took part in the Delphi survey. Although all participants in the Delphi survey were eligible to take part in the consensus meeting, predominantly surgeons (17 out of 24 participants) attended. The next stages to define and agree measures for the CRS will aim to engage a diverse group of stakeholders throughout all stages. An international PPI group of patients with lived experience of bariatric surgery provided input into the different stages of the research. Although patients did not participate in the consensus process, a parallel consensus project on QOL measures for obesity treatments includes patient participants and will be incorporated within the CRS.

The development of a core, minimum set of data items to be collected is the first step of a process attempting to unify international bariatric surgery registry efforts, maximizing the potential of the collected data. All bariatric surgery registries worldwide should be updated with the CRS. The CRS will also provide the opportunity for new national registries to be developed that will align with international efforts. Work now needs to be undertaken to define and select appropriate measures and timepoints for the CRS including the incorporation of QOL measures being defined by the SQOT initiative. This work should be undertaken in parallel for the bariatric surgery research COS to enhance the possibility that data can be combined and compared from both bariatric surgery trials and registries [53]. Alongside the CRS, appropriate data validation processes also need to be embedded within national registries to ensure data collected is of high quality.

## Supplementary Information

Below is the link to the electronic supplementary material.

Supplementary file1 (DOCX 105 KB)

Supplementary file2 (PDF 1096 KB)

Supplementary file3 (PDF 816 KB)

Supplementary file4 (DOCX 38 KB)
