# Supplementary material for: Development of a Bariatric Surgery Core Data Set for an International Registry
Source: Obes Surg. 2023 Mar 24;33(5):1463–75. doi: 10.1007/s11695-023-06545-y (PMC10156789; doi:10.1007/s11695-023-06545-y)
Supplement: Supplementary file 1 — Supplementary file1 (DOCX 105 KB) [file 11695_2023_6545_MOESM1_ESM.docx]

**Developing international core sets for the monitoring and evaluation of bariatric surgery**

**Investigators** (in alphabetical order - final order TBD)**:**

Kerry Avery^1,2,^*, Jane Blazeby^1,2,3^, Wendy Brown^4^, Katy Chalmers^1,2^, Karen Coulman^1,2,5^, John Dixon^4^, Lilian Kow^4^, Ronald Liem^4^, Johan Ottosson^4^, Richard Welbourn^4^

^1^ National Institute for Health Research Bristol Biomedical Research Centre

^2^ Bristol Centre for Surgical Research, Bristol Medical School: Population Health Sciences, University of Bristol, 39 Whatley Road, Bristol, BS8 2PS, UK

**^3^** Division of Surgery, University Hospitals Bristol NHS Foundation Trust, Bristol, BS2 8HW

^4^ International Federation of Surgery for Obesity and Metabolic Disorders (IFSO), 80121 Naples, Italy.

^5^ Obesity and Bariatric Surgery service, North Bristol NHS Trust, Bristol, BS10 5NB, UK

* Correspondence: kerry.avery@bristol.ac.uk

**Funding acknowledgement**

This study is funded by the International Federation for the Surgery of Obesity and Metabolic Disorders (IFSO) and supported by the Royal College of Surgeons of England Bristol Surgical Trials Centre and the NIHR Biomedical Research Centre at University Hospitals Bristol NHS Foundation Trust and the University of Bristol.

# Background

## Bariatric surgery

Obesity rates have tripled since 1975 according to the World Health Organisation.^1^ Within the last three years alone, obesity rates have increased in the adult population by 3% in both the UK (29%) and Australia (31%).^2^ The highest rates of obesity are found in the Pacific islands, the Middle East, North America, the Caribbean Islands and parts of Central and South America.^2^ Obesity is associated with an increased risk of type 2 diabetes, cardiovascular disease, certain cancers, and premature death.^3 4^ Within this population, people with severe and complex obesity (BMI ≥40 kg/m2, or 35-40 kg/m2 with another significant health problem that could be improved by weight loss) suffer the greatest health burdens and are at the highest risk of premature death.^5 6^

Bariatric surgery, combined with behaviour change and dietary management, is the most clinically effective treatment for people with severe and complex obesity, in terms of weight loss and the improvement of co-morbidities such as type 2 diabetes.^6-8^ Recent international data indicate that sleeve gastrectomy (SG, 46.0%) and Roux-en-Y gastric bypass (RYGB, 38.2%) are the most common bariatric operations worldwide with adjustable gastric band (AGB, 5.0%) decreasing in recent years, and the one-anastomosis gastric bypass (OAGB, 7.6%) now gaining popularity.^9^ Each of these procedures works slightly differently; mechanisms include restriction in the amount of food able to be consumed, reduction in hunger, improvement in satiety, shift in food preferences, as well as altered gut hormones, bile acids, and vagal signalling.^10^ However, there are very few well designed and conducted randomised controlled trials with long-term follow-up comparing the effectiveness and safety of the different types of operations. Published studies in this area also suffer from problems with heterogeneity of outcome reporting, making comparison of data from different studies difficult.^7 11 12^ To help address these issues, a core outcome set to be used in effectiveness trials of bariatric surgery was previously developed, containing nine outcomes.^13^ Current work is ongoing through the SQOT (Standardizing Quality of life measures in Obesity Treatment) initiative to determine the most appropriate measures for health-related quality of life (HRQL) – one of the core outcomes identified.^14 15^ Two of the study investigators (RL and KCo) are involved in this initiative.

## Prospective registries

Although RCTs are considered the gold standard study design to compare interventions, they can be difficult and expensive to undertake.^16^ Large prospectively collected registries can make an important contribution to the understanding of the longer-term effects of the different types of surgery, by allowing the inclusion of data from a large number of patients on a national level.^16-18^ Registries may be used to examine disease epidemiology, treatment effectiveness and the quality of patient care. Data recorded in registries may include demographic, diagnostic, prognostic or technical variables, as well as clinically important outcomes, including both effectiveness outcomes and complication rates for surgical interventions.^18^ There are 17 national bariatric surgery registries in existence, however not all have been developed with key quality indicators defined at the outset, and as with published research studies in this area, variables measured in different registries, including their timing of measurement vary considerably between countries, making cross-country comparisons difficult.^2 18^ Previous work has been undertaken to amalgamate registry data from the Netherlands, Sweden and Norway to compare outcome data for bariatric surgery.^17 19^ The International Federation for the Surgery of Obesity and Metabolic Disorders (IFSO) has set up a global registry project to allow for comparisons of obesity and bariatric surgery on an international level with the ultimate aim of improving patient care, however at present it includes mainly demographic data with limited 12-month follow-up outcomes.^2 9 18 20^ An agreed standardised set of variables (core sets) that should be measured and reported as a minimum in all evaluations, is needed to enable the comparison of bariatric surgery registry data on an international level.^2^ Additionally, the timing of follow-up measurement also requires standardisation to ensure comparability of short, medium and long-term outcomes of bariatric surgery.

# Aims and objectives

The aim of this project is to develop international core sets for the standardised and efficient monitoring and evaluation of bariatric surgery.

## Objectives

The specific objectives are:

1. To develop a comprehensive list of variables relevant to the monitoring and evaluation of bariatric surgery from multiple international data sources (and where appropriate identify appropriate measures);
2. To categorise the variables for inclusion into one of three core sets representing the different phases of bariatric surgery registry data collection (baseline data, procedure information and surgical complications, effectiveness outcomes);
3. To scientifically prioritise the identified variables with key stakeholders, including the timepoints at which surgical complications and effectiveness outcomes should be measured;
4. To nest a methodological project to optimise methods to reach consensus within core set development.

# Stakeholder involvement

A Study Management Group (SMG) will be convened to oversee all phases of the study, with representation from a diverse group of international stakeholders including relevant members of the bariatric multidisciplinary team (MDT), research methodologists, and members of the IFSO Registry Executive committee. This will include a maximum of 12 professionals. Current members include Kerry Avery, Jane Blazeby, Wendy Brown, Karen Coulman, Katy Chalmers, John Dixon, Lilian Kow, Ronald Liem, Johan Ottosson and Richard Welbourn. A separate Patient and Public Involvement (PPI) group consisting of 4-6 members will be formed to provide guidance on the different phases of the project. This group will meet separately to the professional SMG to ensure patient representatives are able to adequately express their views (see more under ‘Patient and Public Involvement’).

# Scope of the core sets

The core sets are intended for use in the monitoring and evaluation of bariatric surgery including national and international bariatric surgery registries, audit and research. Use of the core sets will facilitate the standardised measurement and reporting of a minimum set of data relevant to support the evaluation of bariatric surgery for the purposes of routine clinical practice, audit and research.

# Methods

The core sets will be developed according to principles outlined in the Core Outcome Measures for Effectiveness Trials (COMET) Handbook and Core Outcome Set-Standards for Development (COS-STAD) guidelines.^21 22^ The study will comprise three phases.

## Phase 1: Development of the long list of variables

A comprehensive long list of potentially relevant variables to include in the core sets will be generated from the following data sources:

1. Variables identified during the development of an existing COS for bariatric surgery effectiveness trials in the BARIACT study.^13^ Outcomes included in the BARIACT study were identified from three systematic reviews^11 12 23^ and qualitative interviews with patients that had undergone bariatric surgery^24^, the methods for which have previously been reported in full.^13 25^
2. Variables identified from a Dutch data dictionary project, which collated variables from 11 existing national bariatric surgery registries from the following countries: Australia & New Zealand, Austria, Brazil, Kuwait, Mexico, the Netherlands, Norway & Sweden, Russia, Turkey, UK and USA (data to be published soon).
3. Variables identified from systematic searches of bariatric surgery effectiveness trials covering the years 2013-2020 (unpublished data) to inform the By-Band-Sleeve study.

Variables identified from each data source will be combined into a single long list, and any duplicates removed or overlapping items combined by the study team, which will include methodologists and health professionals (KCh, KCo, KA, JB). Expert bariatric surgery health professionals from the SMG and patients who have undergone bariatric surgery (see ‘Patient and Public Involvement’) will review the list to ensure no important variables have been missed. The long list of variables will be mapped into broader domains where relevant. The study team will then group the long list of variables/domains into three lists representing the following categories:

1. Baseline data (e.g. variables that will only be measured at baseline, such as demographics);
2. Surgical procedure information (measured only once peri-operatively) and surgical complications (measured during and/or after surgery);
3. Effectiveness outcomes (will be measured at baseline and follow-up).

## Phase 2: Prioritisation of variables in a Delphi survey

Agreement on the variables to be included in each core set (and the timings of follow-up measurement of the effectiveness outcomes core set) will be reached through a sequential, two-round online Delphi survey (phase 2) followed by a face-to-face/virtual consensus meeting (phase 3). Strengths of the Delphi process are that it allows for a diverse group of participants from a wide geographical area to participate, while preserving anonymity so as to prevent results from being strongly influenced by the views of dominating individuals.^26^

Each variable/domain included in the final long list described above will be formatted into an item for the Delphi survey questionnaire. Each item will be accompanied by a nine-point Likert scale for rating the importance of including the variable in the final core sets, labelled 1 to 3 ‘not important’, 4 to 6 ‘important but not critical’ and 7 to 9 ‘critical’, based on the Grading of Recommendations Assessment, Development and Evaluation (GRADE) guidelines.^27^ Additional free text items will be included to enable stakeholders to propose new variables. Proposed new variables recommended by two or more participants will be considered for inclusion in the next survey round by the study team.^28^ Additional survey items will be included asking stakeholders to prioritise follow-up timepoints for outcome data collection for the effectiveness outcomes core set. Potential timepoints will be informed by the data sources described above and discussion with the SMG. Survey questionnaires will be reviewed by the SMG to ensure clarity and acceptability.

### Identification of stakeholder participants

A broad range of specialist health professionals involved in the care of bariatric surgery patients (e.g. surgeons, physicians, dietitians, nurses, psychologists, and anaesthetists) will be invited to take part in the survey. An email invitation to participate in the Delphi survey will be sent to all IFSO members from the IFSO president. Email invitations will also be sent to members of the 66 official member societies of IFSO, from each participating member society president.

### Delphi survey rounds

Stakeholders will be invited to participate in two sequential survey rounds, administered online using REDCap electronic data capture tools hosted at the University of Bristol ^29 30^ in accordance with CHERRIES guidelines for electronic surveys.^31^

### Round 1

Participants will be asked to rate the importance of variables on a nine-point scale ranging from one (not important) to nine (very important). Descriptive statistics will be used to summarise the results of round one, including the number of participants rating each outcome 7-9 (critical). The median score for each item will be calculated and used to provide feedback for the round 2 questionnaire (see next section on Round 2 for more information). All statistical analyses will be undertaken using STATA 15 statistical software programme.^32^

### Round 2

All respondents to the round 1 questionnaire will be invited to complete round 2. The round 2 questionnaire will be identical to round 1 but will also include personalised feedback from round 1. Participants will receive their own individual round 1 scores for each item, the median scores of their peer group, of other health professionals (excluding their peer group), and of the whole group. Participants will be asked to re-rate the items on the questionnaire, considering the round 1 feedback. Items that are rated 7-9 by ≥70% of participants AND 1-3 by <15% of participants will be taken forward for discussion and voting at a consensus meeting. All other items will not be carried forward. These limits were selected based on previous studies using consensus methods to develop core outcome sets.^33 34^

## Phase 3: Consensus meeting

In phase 3, a stakeholder consensus meeting will be held, either face-to-face or virtually, to agree and ratify the final core sets.

### Stakeholders

Purposive sampling will be used to ensure that all relevant health professionals involved in the care of patients undergoing bariatric surgery (as specified above for the Delphi survey) are represented within the consensus meeting. Purposive sampling involves deliberately selecting participants based on their characteristics in order to enable a detailed exploration of the research question.^35 36^ This is in contrast to the statistical sampling used in RCTs, which aims to create a statistically representative sample, so that study findings can be generalised to the larger population.^35 36^ As the aim of this study is to develop consensus on the variables to be included in core sets, rather than detect differences in outcomes between intervention groups (as would be undertaken in an RCT), purposive sampling is appropriate.

Although patients will not take part in the consensus meeting, the PPI group will be consulted in advance of the consensus meeting to discuss the items being taking forward to the consensus meeting and ensure important items have not been missed.

#### Conduct of the meeting, data collection, and analysis

The consensus meeting will take place prior to the IFSO conference in Miami in July 2021. The meeting will comprise two parts, facilitated by a chairperson to enable moderated discussion.

##### Part 1:

The meeting will begin with a description of the study, its aims and methods, and work undertaken to date. Participants will be presented with a summary of the Delphi survey results and provided with three lists (corresponding with the three core sets to be developed) of the items (variables) that require voting during the consensus meeting. The wording of items may be shortened and simplified for the consensus meeting so that items are easy to read and understand on slides prepared in Microsoft PowerPoint.^37^ The meeting format will allow for further discussion and clarification of items as needed. Participants will be asked to spend a few minutes looking through the list and putting a star next to their top five items in each section. Participants will not hand in their lists; the purpose of the lists is simply to enable participants to begin thinking about the prioritisation of items to aid with subsequent voting.

##### Part 2:

The Chair will begin by describing the online voting process using TurningPoint web software.^38^ Each of the items to be voted on will be presented on a separate slide in Microsoft PowerPoint. Further description and clarification will be given for items as needed. The percentage of participants who rated the item 7-9 in the final round of the survey will also be included on each slide for reference. Using anonymised, real-time voting in TurningPoint web, participants will vote ‘In’ or ‘Out’ to indicate whether they feel each item should be included in the final core sets. Once all participants have cast their votes, a histogram and descriptive statistics will be presented to the group for immediate feedback. Items will be retained for the core sets if ≥70% of participants vote ‘Yes’ AND <15% vote ‘No’, and items discarded if ≥70% vote ‘No’, and <15% vote ‘Yes’. For items with bimodal distributions (similar number of participants voting ‘In’ or ‘Out’), the item will be carried forward to a second round of voting which will include further discussion to understand the reasons behind the voting in the first round. The same criteria will be applied to the analysis of the voting, and further rounds of voting and discussion will continue until consensus has been reached on the final core sets.

# Sample size

There is no agreed methodology for determining the sample size required for consensus processes to develop a core set. Sample size is dependent on the scope of a core set and decisions on the stakeholder groups to be involved, as well as practical feasibility considerations.^21 26^ The UK-based BARIACT project, which developed a COS for bariatric surgery effectiveness trials, included 168 health professional and 90 patient Delphi survey respondents, 33 participants in the professional consensus meeting, and eight participants in the patient consensus meeting.^13^ As the current study will include health professionals only and on an international level, the target sample size is for 500 respondents to the Delphi survey, and a maximum of 35 participants in the consensus meeting.

This project will be registered on the COMET (Core Outcome Measures in Effectiveness Trials) database.^39^

# Embedded methodological study

To explore optimal methods for providing feedback to encourage prioritisation between Delphi survey rounds, participants will be randomised to receive one of two versions of the round 2 questionnaire. The two versions will differ only in the type of feedback about the round 1 results provided. In version A of the questionnaire (standard feedback), the feedback will include only the standard feedback of the round 1 results (detailed above). In version B (enhanced feedback), the questionnaire will also include a preceding paragraph detailing the top 5 items for each of the three core sets. Participants will be randomly allocated to one of the two groups and blinded to their group allocation. The results from round 1 (e.g. the number and types of items that are prioritised) will be compared between the two allocation groups.

# Patient and Public Involvement

An international group of 4-6 patients who have undergone bariatric surgery will be formed to advise on all phases of the study. In particular, the group will be consulted to ensure that no important variables are missing from the long list of variables used to develop the Delphi survey, and those brought forward to the consensus meeting, as well as the timepoints of effectiveness outcomes data collection to be surveyed in the Delphi process. There is strong patient involvement in the international SQOT initiative which is working to standardize quality of life measures in obesity treatment.^15^ The SQOT initiative is being undertaken in parallel to this project (which is focusing on clinical and surgical outcomes) and will feed into the results of this project.

# Ethical approval

Ethical approval for the study will be sought from the University of Bristol Health Sciences Research Ethics Committee. Completion of the Delphi survey will be taken as implying informed consent to participate in the study.

# Dissemination

Details, including findings, of this study will be published in a relevant peer reviewed scientific journal(s). Findings will also be disseminated at relevant clinical and methodological conferences and through the IFSO website.

# References

1. World Health Organisation. Obesity and overweight. 2018 [Available from: <https://www.who.int/en/news-room/fact-sheets/detail/obesity-and-overweight>.

2. Ramos A, Kow L, Brown W, et al. 5th IFSO Global Registry Report. Reading: Dendrite Clinical Systems Ltd, 2019.

3. Must A, Spadano J, Coakley EH, et al. The disease burden associated with overweight and obesity. *JAMA* 1999;282(16):1523-29.

4. Wang Y, McPherson K, Marsh T, et al. Health and economic burden of the projected obesity trends in the USA and the UK. *Lancet* 2011;378(9793):815-25.

5. Dietz WH, Baur LA, Hall K, et al. Management of obesity: improvement of health-care training and systems for prevention and care. *Lancet* 2015;385(9986):2521-33.

6. National Institute for Health and Care Excellence. Obesity: identification, assessment and management of overweight and obesity. Clinical Guideline [CG189]. London, 2014.

7. Colquitt Jill L, Pickett K, Loveman E, et al. Surgery for weight loss in adults. *Cochrane Database of Systematic Reviews* 2014(8) doi: 10.1002/14651858.CD003641.pub4

8. O'Brien PE, MacDonald L, Anderson M, et al. Long-term outcomes after bariatric surgery: fifteen-year follow-up of adjustable gastric banding and a systematic review of the bariatric surgical literature. *Ann Surg* 2013;257(1):87-94.

9. Welbourn R, Hollyman M, Kinsman R, et al. Bariatric Surgery Worldwide: Baseline Demographic Description and One-Year Outcomes from the Fourth IFSO Global Registry Report 2018. *Obes Surg* 2019;29(3):782-95.

10. Miras AD, le Roux CW. Mechanisms underlying weight loss after bariatric surgery. *Nature reviews Gastroenterology & hepatology* 2013;10(10):575-84.

11. Hopkins JC, Howes, N., Chalmers, K., Savovic, J., Whale, K., Coulman, K.D., Welbourn, R., Whistance, R.N., Andrews, R.C., Byrne, J.P., Mahon, D., Blazeby, J.M. Outcome reporting in bariatric surgery: an in-depth analysis to inform the development of a core outcome set, the BARIACT Study. *Obesity Reviews* 2015;16(1):88-106.

12. Coulman KD, Abdelrahman T, Owen-Smith A, et al. Patient-reported outcomes in bariatric surgery: a systematic review of standards of reporting. *Obesity Reviews* 2013;14(9):707-20.

13. Coulman KD, Hopkins J, Brookes ST, et al. A Core Outcome Set for the Benefits and Adverse Events of Bariatric and Metabolic Surgery: The BARIACT Project. *PLoS Med* 2016;13(11):e1002187.

14. de Vries CEE, Kalff MC, Prinsen CAC, et al. Recommendations on the most suitable quality-of-life measurement instruments for bariatric and body contouring surgery: a systematic review. *Obes Rev* 2018;19(10):1395-411.

15. SQOT initiative: Standardizing Quality of Life measures in Obesity Treatment 2020 [Available from: <https://sqotinitiative.wixsite.com/sqot>. Accessed 26.2.20/

16. Camm AJ, Fox KAA. Strengths and weaknesses of 'real-world' studies involving non-vitamin K antagonist oral anticoagulants. *Open heart* 2018;5(1):e000788.

17. Poelemeijer YQM, Liem RSL, Vage V, et al. Perioperative Outcomes of Primary Bariatric Surgery in North-Western Europe: a Pooled Multinational Registry Analysis. *Obes Surg* 2018;28(12):3916-22.

18. Brown WA, MacCormick AD, McNeil JJ, et al. Bariatric Surgery Registries: Can They Contribute to Improved Outcomes? *Current obesity reports* 2017;6(4):414-19.

19. Poelemeijer YQM, Liem RSL, Vage V, et al. Gastric Bypass Versus Sleeve Gastrectomy: Patient Selection and Short-term Outcome of 47,101 Primary Operations from the Swedish, Norwegian, and Dutch National Quality Registries. *Ann Surg* 2019.

20. Welbourn R, Pournaras DJ, Dixon J, et al. Bariatric Surgery Worldwide: Baseline Demographic Description and One-Year Outcomes from the Second IFSO Global Registry Report 2013-2015. *Obes Surg* 2018;28(2):313-22.

21. Williamson PR, Altman DG, Bagley H, et al. The COMET Handbook: version 1.0. *Trials* 2017;18(Suppl 3):280.

22. Kirkham JJ, Davis K, Altman DG, et al. Core Outcome Set-STAndards for Development: The COS-STAD recommendations. *PLoS Med* 2017;14(11):e1002447.

23. Coulman KD, MacKichan F, Blazeby JM, et al. Patient experiences of outcomes of bariatric surgery: a systematic review and qualitative synthesis. *Obes Rev* 2017;18(5):547-59.

24. Coulman KD, MacKichan F, Blazeby JM, et al. Patients’ experiences of life after bariatric surgery and follow-up care: a qualitative study. *BMJ Open* 2020;10(2):e035013.

25. Coulman KD, Howes N, Hopkins J, et al. A Comparison of Health Professionals' and Patients' Views of the Importance of Outcomes of Bariatric Surgery. *Obes Surg* 2016;26(11):2738-46.

26. Sinha IP, Smyth RL, Williamson PR. Using the Delphi Technique to Determine Which Outcomes to Measure in Clinical Trials: Recommendations for the Future Based on a Systematic Review of Existing Studies. *Plos Medicine* 2011;8(1)

27. Guyatt GH, Oxman AD, Kunz R, et al. GRADE guidelines: 2. Framing the question and deciding on important outcomes. *J Clin Epidemiol* 2011;64(4):395-400.

28. Harding AJE, Morbey H, Ahmed F, et al. Developing a core outcome set for people living with dementia at home in their neighbourhoods and communities: study protocol for use in the evaluation of non-pharmacological community-based health and social care interventions. *Trials* 2018;19(1):247.

29. Harris PA, Taylor R, Minor BL, et al. The REDCap consortium: Building an international community of software platform partners. *Journal of biomedical informatics* 2019;95:103208.

30. Harris PA, Taylor R, Thielke R, et al. Research electronic data capture (REDCap)--a metadata-driven methodology and workflow process for providing translational research informatics support. *Journal of biomedical informatics* 2009;42(2):377-81.

31. Eysenbach G. Improving the quality of Web surveys: the Checklist for Reporting Results of Internet E-Surveys (CHERRIES). *Journal of medical Internet research* 2004;6(3):e34.

32. Stata/MP 15.1. College Station, Texas: StataCorp, 2017.

33. Avery KNL, Chalmers KA, Brookes ST, et al. Development of a Core Outcome Set for Clinical Effectiveness Trials in Esophageal Cancer Resection Surgery. 2018;267(4):700-10.

34. McNair AG, Whistance RN, Forsythe RO, et al. Core Outcomes for Colorectal Cancer Surgery: A Consensus Study. *PLoS Med* 2016;13(8):e1002071.

35. Carter S, Henderson L. Approaches to qualitative data collection in social science. In: Bowling A, Ebrahim S, eds. Handbook of Health Research Methods: Investigation, Measurement and Analysis. Maidenhead, Berkshire: Open University Press 2005:215-29.

36. Ritchie J, Lewis J, Elam G. Designing and Selecting Samples. In: Ritchie J, Lewis J, eds. Qualitative Research Practice: A Guide for Social Science Students and Researchers. London: SAGE Publications Ltd, 2003.

37. Microsoft PowerPoint 2013. Redmond, Washington: Microsoft.

38. TurningPoint web: Turning Technologies, LLC, 2020.

39. University of Liverpool. COMET Initiative: <http://www.comet-initiative.org/> Accessed: 26.2.2020
