# Supplementary material for: Development of a Bariatric Surgery Core Data Set for an International Registry
Source: Obes Surg. 2023 Mar 24;33(5):1463–75. doi: 10.1007/s11695-023-06545-y (PMC10156789; doi:10.1007/s11695-023-06545-y)
Supplement: Supplementary file 2 — Supplementary file2 (PDF 1096 KB) [file 11695_2023_6545_MOESM2_ESM.pdf]

Developing international core sets for the monitoring and evaluation of bariatric surgery:

Consensus meeting

Information Pack

CONFIDENTIAL

Contents

1. Meeting objective

2. Provisional agenda

3. What are 'core sets' and why do we need them for the IFSO Global Registry?

4. Previous steps in this core sets project

5. Three core sets proposed

6. Items meeting Delphi consensus threshold

7. Items not meeting Delphi consensus threshold

8. Which items are critically important for the core sets?

1. Meeting objective

Thank you for agreeing to participate in the virtual consensus meeting to develop core sets for the IFSO Global Registry. This information pack summarises key results from the previous steps in this project and gives you an idea of how the consensus meeting will run. Please take some time to read through the material in advance of the meeting. This will give you a chance to gather your thoughts ahead of time, which will help with discussions on the day of the meeting. The objective of the meeting is to agree on what items should be included in core sets for a global bariatric surgery registry. To do this, we plan to run the meeting using the provisional timetable detailed in section 2.

2. Provisional agenda

The meeting will start at: 11am GMT, 12 pm CET, 6am EST, 10pm AEDT. You will be able to join the meeting from 10 minutes before the start time.

Time (GMT)	Activity
10:50am	**Join the online meeting**
11:00am	**Introduction**<br>• Welcome from the meeting Chair (Prof Jamie Kirkham)<br>• Overview of the IFSO Global Registry Project including scope of core sets project (Prof Wendy Brown)<br>• Instructions for how the meeting will run and questions (Dr Kerry Avery/Dr Karen Coulman)
11:10am	**Core set 1 ('Baseline only information'):**<br>• Discussion and voting on items rated 'critical importance' in the Delphi survey by 95% or more of participants<br>• Small group discussion to select the top 3 of remaining items rated 'critical importance' by 70% or more of participants<br>• Break while results compiled<br>• Further discussion and voting in whole group
11:45am	**Core set 2 ('Effectiveness outcomes'):**<br>• Discussion and voting on items rated 'critical importance' in the Delphi survey by 95% or more of participants<br>• Small group discussion to select the top 3 of remaining items rated 'critical importance' by 70% or more of participants<br>• Break while results compiled<br>• Further discussion and voting in whole group
12:20pm	**Core set 3b ('Potential complications and side-effects of surgery'):**<br>• Discussion and voting on items rated 'critical importance' in the Delphi survey by 95% or more of participants<br>• Small group discussion to select the top 3 of remaining items rated 'critical importance' by 70% or more of participants<br>• Break while results compiled<br>• Further discussion and voting in whole group
12:55pm	**Core set 3a ('Surgical procedure information' – surgeons only, break for others):**<br>• Discussion and voting on items rated 'critical importance' in the Delphi survey by 95% or more of participants

	• Small group discussion to select the top 3 of remaining items rated 'critical importance' by 70% or more of participants<br>• Break while results compiled<br>• Further discussion and voting in whole group
1:30pm	**Break**
1:40pm	**Discussion and finalisation of the full core set**
2:00pm	**Close of meeting**

Zoom link to join meeting

https://bristol-ac-uk.zoom.us/j/91377141862?pwd=cktpU3R5a0t1UEJaUXRqYWJJeTdBQT09

Meeting ID: 913 7714 1862
Passcode: 724353

Additional dial-in details if dialling in from your telephone will be available in the Calendar invite which you will receive over email.

3. What are 'core sets' and why do we need them for the IFSO Global Registry?

The International Federation for the Surgery of Obesity and Metabolic Disorders (IFSO) has set up a global registry project to allow for monitoring and comparisons of obesity and bariatric surgery on an international level, with the aim of improving patient care. Using agreed standardised sets of information, also known as '**core sets'**, will allow for data between registries to be compared and combined within the IFSO Global Registry more easily. A core set includes the information that should be **measured and reported *as a minimum* in all evaluations of a particular intervention**. Using core sets does not stop individual registries from collecting other items they consider important. To be consistent across registries, however, at least the items in the core sets should be measured alongside any other items. In this meeting we aim to produce core sets for the IFSO global registry. There are a lot of different items collected in bariatric surgery registries and many of these could be considered 'important', but the items in the core sets will be those we agree are 'critically important'.

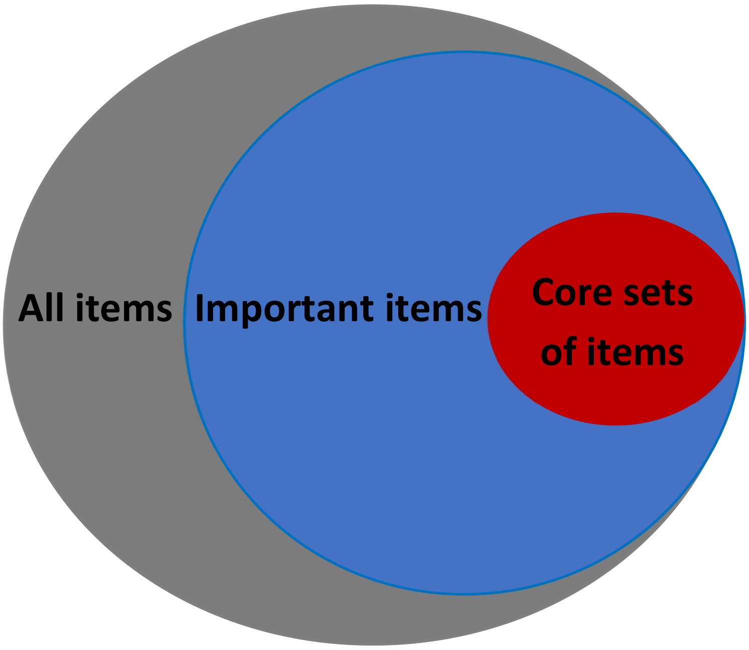

In this first stage of creating core sets, we need to agree (reach consensus) on 'what' to measure. We will not decide on 'how' to measure each item at this stage – this will require separate work to agree the best way to measure all the specific items in the core sets. The meeting also will not involve deciding on 'when' each item should be measured, as that falls within the area of 'how' to measure. The output from this meeting is essential in helping to direct that future work, as we need to know 'what' to measure before we can think about 'how' to measure it.

4. Previous steps in this core sets project

There were two main steps in the project that have led up to the consensus meeting which you are participating in today.

1. We first compiled a comprehensive long list of all potentially important items to include in a bariatric surgery registry. We used:

    a. Items identified in the development of an existing core set for bariatric surgery effectiveness trials in the BARIACT study.[1] This was developed with patient input, the full methods for the study are reported here.

    b. Items identified from a Dutch data dictionary project, which collated items from 11 existing national bariatric surgery registries.[2]

    c. Items identified from systematic searches of bariatric surgery effectiveness trials between 2013-2020 (unpublished data) to inform the By-Band-Sleeve study.[3]

    d. Additional items deemed of importance to patients contributed by an international patient advisory group

2. We undertook an international Delphi survey of IFSO members. This two-round online questionnaire asked participants to rate (on a scale of 1-9) the importance of including each item within a bariatric surgery registry. A score of 7-9 indicated an item of critical importance, 4-6 an item that is important but not critical, and 1-3 an item of limited importance. 272 IFSO members took part in round 1, and 133 in round 2, representing a range of disciplines including surgeons, dietitians, psychologists, specialist nurses, physicians, and other professionals. The patient advisory group commented on the results of Delphi survey (see section 7 for more information on their feedback).

    Items rated 7-9 by 70% or more of participants in the Delphi survey met the threshold for being automatically brought forward to this consensus meeting. This threshold was set before the Delphi survey began. While we will consider the Delphi survey results to guide our discussion at the consensus meeting, it is during the consensus meeting that the final core sets will be decided.

References

1. Coulman KD, Hopkins J, Brookes ST, Chalmers K, Main B, Owen-Smith A, et al (+11). A Core Outcome Set for the Benefits and Adverse Events of Bariatric and Metabolic Surgery: The BARIACT Project. *PLoS Med* 2016;13(11):e1002187.

2. Akpinar EO, Marang- van de Mheen PJ, Nienhuijs SW, Greve JWM, Liem RSL. National Bariatric Surgery Registries: an International Comparison. *Obes Surg* 2021;31(7):3031-9.

3. Rogers CA, Reeves BC, Byrne J, Donovan JL, Mazza G, Paramasivan P, et al (+6). Adaptation of By-Band randomized clinical trial to By-Band-Sleeve to include a new intervention and maintain relevance of the study to practice. *Br J Surg* 2017;104(9):1207-14.

5. Three core sets proposed

We are proposing 3 core sets, each representing a different time point at which information is collected in a bariatric surgery registry.

- **Core set 1 (Baseline Information)** will focus on information that is collected *only once before* surgery

- **Core set 2 (Effectiveness Outcomes)** will focus on information used to measure the impact or effectiveness of bariatric surgery - measured *both before and after* surgery

- **Core set 3 (Surgical Information)** will focus on information measured *during or after* surgery, including information about the surgical procedure **(Set 3a - surgeons only)** and potential complications and side-effects of surgery **(Set 3b)**

In the next section we show you the **72 items** from the Delphi survey that met the threshold to be automatically included for discussion at the consensus meeting. All items that have reached this stage are generally agreed to be very important, but a core set should include only the critically important items. 72 items are a lot to include within the core sets, and we will aim to reduce this to a more feasible number during the consensus meeting. **We need your help to decide which items are critically important to include.**

6. Items meeting Delphi consensus threshold

The tables in this section show the 72 items from the Delphi survey that met the threshold to be automatically included for discussion at the consensus meeting. They are presented in descending order according to the percentage of all participants scoring 7-9. The percentage scoring 7-9 within stakeholder groups is also presented (surgeons and other health professionals). Due to smaller numbers of other health professionals (dietitians, nurses, physicians, psychologists, and others) who participated in the Delphi survey, these groups have been combined.

- Scores in dark green indicate where an item was rated 7-9 by **95% or more** of participants.
- Scores in light green indicate where an item was rated 7-9 by **between 70 and 95%** of participants.
- Scores in red indicate where **less than 70% of one group only** rated the item 7-9.

<u>Table 1: Core Set 1 – Baseline Information</u> (information that is collected *<u>only once before</u>* surgery)

**Item (n=10)***	**Percentage scoring 7-9**		
	**Surgeons (n=68)**	**Other HCPs (n=55)**	**All (n=133)**
History of any previous bariatric surgery	97.1	94.4	95.9
Height of the patient	88.2	88.9	88.5
Other medical conditions not directly related to obesity e.g., type 1 diabetes, dementia	92.6	81.5	87.7
Age of patient	82.4	87.0	84.4
Medication history	86.8	81.5	84.4
History of any previous abdominal surgery (other than bariatric surgery)	80.9	75.9	78.7
Weight history	74.6	81.5	77.7
Sex of patient	77.9	75.9	77.0
Time period over which any pre-surgery weight loss occurred	70.1	72.2	71.1
Which members of MDT involved	64.2	77.8	70.2

*Items where 70% or more of ALL participants combined scored the item 7-9

Table 2: Core set 2 - Effectiveness Outcomes (information used to measure the impact or effectiveness of bariatric surgery - measured *both before and after* surgery)

Item (n=23)*	Percentage scoring 7-9		
	Surgeons (n=68)	Other HCPs (n=55)	All (n=133)
Diagnosis of Type 2 diabetes	98.5	96.0	97.5
Weight	97.0	96.2	96.6
Addictive behaviours, e.g., alcohol, gambling, illicit drugs	92.5	96.2	94.1
Medication for Type 2 diabetes	97.1	90.0	94.1
Problems with breathing during sleep (obstructive sleep apnoea)	94.1	94.0	94.1
Long standing acid reflux, or use of medication (gastro-esophageal reflux or GERD)	92.5	96.0	94.0
Depression, or use of medication	92.5	92.3	92.4
Alcohol	89.6	94.2	91.6
Suicidal thoughts	91.0	90.4	90.8
Smoking	87.9	94.2	90.7
Binge eating	85.1	96.2	89.9
High blood pressure, or use of blood pressure medication (hypertension)	88.2	90.0	89.0
Obesity-related liver disease, e.g., non-alcoholic fatty liver disease	88.1	90.0	88.9
Congestive heart failure, or use of medication	85.3	89.8	87.2
Elevated fat and cholesterol in the blood, or use of medication (dyslipidemia)	85.3	80.0	83.1
Risk of future heart and vascular problems (assessment of cardiovascular risk)	76.5	85.7	80.3
Feelings towards one's body shape or appearance (body dysmorphia/ dysmorphic disorder)	71.6	86.5	78.2
Physical activity levels	74.6	82.7	78.2
Abnormal or irregular heartbeat, or use of medication (arrhythmia)	77.9	78.0	78.0

Male or female reproductive function, e.g., polycystic ovary syndrome	70.1	82.7	75.6
Use of weight loss medication	74.6	76.0	75.2
Joint disease, or use of medication, or being considered for joint replacement	70.6	80.0	74.6
Body shape, e.g., waist and hip measurements	67.2	76.5	71.2

*Items where 70% or more of ALL participants combined scored the item 7-9.

Table 3: Core set 3a (Surgeons only) - Information about the surgical procedure (measured *during* surgery)

Item (n=14)*	Percentage scoring 7-9
	Surgeons (n=68)
Measurements of limb length (not for sleeve gastrectomy)	98.5
Name of surgical procedure, e.g., sleeve gastrectomy, one-anastomosis gastric bypass	97.0
Closure of hernia defects undertaken (not for sleeve gastrectomy)	97.0
Surgical approach to gain access, e.g., laparoscopic, open or endoscopic	95.5
Duration of balloon implantation (when removed)	91.9
Hiatus hernia repair undertaken	90.9
Fill volume of balloon	88.5
Pre-operative assessment of surgical risk, e.g., OS-MRS score or similar	87.9
Type/make of device (including band and balloon, adjustable or non-adjustable)	87.3
Size of bougie	86.4
Method of balloon placement, e.g., swallowed or endoscopically placed	83.6
Distance between resection and pylorus (for sleeve gastrectomy only)	83.1
Height of staples used	74.2
Type of reinforcement used	73.8

*Items where 70% or more of surgeons scored the item 7-9

Table 4: Core set 3b - Potential complications and side-effects of surgery (measured *during* or *after* surgery)

Item (n=25)*	Percentage scoring 7-9		
	Surgeons (n=68)	Other HCPs (n=55)	All (n=133)
Clinical malnutrition	98.5	98.0	98.3
Whether a re-intervention occurred, including a classification of its severity, e.g., Clavien-Dindo or similar	98.5	97.9	98.2
Bleeding inside the body (intra-abdominal or endoluminal)	98.5	96.0	97.4
Problems with the heart, vessels, or blood clots (cardiovascular problems or venous thromboembolism)	98.5	96.0	97.4
Problems with anastomotic/staple line/suture line including subsequent infections	100.0	93.9	97.4
Death from surgical complications whilst still in hospital (in-hospital mortality)	98.5	94.1	96.6
Vitamin and mineral levels	95.4	98.0	96.6
Obstruction including ileus and/or hernia	98.5	94.0	96.5
Complications that may occur shortly after the operation when the patient is still in hospital	100.0	92.0	96.5
Complications that occur sometime after the operation, once the patient has been discharged	100.0	92.0	96.5
Accidental damage to other organs during surgery (organ injury)	96.9	96.0	96.5
Death after discharge from hospital (post-discharge mortality)	98.5	90.2	94.8
Cause of death	96.9	92.2	94.8
Problems with the kidneys, including rhabdomyolysis (renal problems)	95.4	92.0	93.9
Problems with gastric and/or stomal ulcers	92.3	96.0	93.9
Unplanned use of high dependency, intensive care or critical care units	93.8	94.0	93.9
Problems swallowing or bringing food back up (dysphagia/regurgitation)	89.2	94.1	91.4
Liver problems	92.2	90.0	91.2

The amount and type of food patients consume (nutritional intake)	81.5	96.1	87.9
Problems with drops in blood sugar after a meal (reactive hypoglycaemia)	83.1	94.0	87.8
When food moves too quickly from the stomach into the small intestine causing symptoms such as cramps, diarrhea, nausea, feeling hot and sweaty (dumping syndrome)	78.5	94.1	85.3
Problems with gallstones	80.0	82.4	81.0
Pain or discomfort in the body	72.3	84.3	77.6
Feeling sick or vomiting (nausea)	73.4	76.5	74.8
Problems in bone strength (bone density)	69.2	80.0	73.9

*Items where 70% or more of ALL participants combined scored the item 7-9

7. Items not meeting Delphi consensus threshold

The items in this section did not reach the threshold to automatically be entered for discussion in the consensus meeting. Please look through the tables in this section and decide whether any of the items deserve to be considered further because you consider them 'critically important' for the core sets. Keep in mind that these items did not meet the threshold of importance set in the Delphi study and that it is important that the core sets do not include too many items, so they are feasible to use. Any item from these tables should only be 'saved' if you feel it is critically important for the core set.

If you would like to 'save' any of these items for discussion at the consensus meeting, please email karen.coulman@bristol.ac.uk by Wednesday 17th November at 5:00pm GMT with the specific items you would like to 'save'. Any items not 'saved' at this stage will not be considered for the core sets.

Table 5: Core Set 1 – Baseline Information (information that is collected *only once before* surgery)

Item (n=7)*	Percentage scoring 7-9		
	Surgeons (n=68)	Other HCPs (n=55)	All (n=133)
Details of previous weight loss programs	58.2	68.5	62.8
Ability of patient to purchase/afford supplements for life, post-surgery**	53.7	69.8	60.8
Source of funding	55.9	53.7	54.9
Duration of type 2 diabetes	50.7	51.9	51.2
Ethnicity of the patient**	38.2	53.7	45.1
Educational level of the patient	33.8	46.3	39.3
Date referred for surgery	40.3	29.6	35.5

*Items where less than 70% of ALL participants combined scored the item 7-9

**Items the international patient advisory group felt were important to save

Table 6: Core set 2 - Effectiveness Outcomes (information used to measure the impact or effectiveness of bariatric surgery - measured *both* *before and after* surgery)

Item (n=10)*	Percentage scoring 7-9		
	Surgeons (n=68)	Other HCPs (n=55)	All (n=133)
Long standing diseases of the lungs such as asthma (chronic pulmonary disease)	66.2	62.0	64.4
Ability to fall asleep at night or quality of sleep (sleep disorders other than sleep apnoea)	58.2	67.3	62.2
Thyroid function, or use of medication (hypothyroidism)	59.7	64.0	61.5
Changes in family and relationship**	46.3	67.3	55.5
Long standing fluid retention (lymphedema)**	31.3	54.0	41.0
Anger management problems	31.3	48.1	38.7
Employment	31.8	46.2	38.1
Abnormal accumulation of fat in legs/arms (lipedema)**	23.9	52.0	35.9
Changes in gut microbiota (gut flora)	20.0	52.0	33.9
Bladder problems (urinary incontinence)	23.9	34.0	28.2

*Items where less than 70% of ALL participants combined scored the item 7-9

**Items the international patient advisory group felt were important to save

Table 7: Core set 3a (Surgeons only) - Information about the surgical procedure (measured *during* surgery)

Item (n=3)*	Percentage scoring 7-9
	Surgeons (n=68)
Length of time spent in hospital after admission for surgery	66.7
Make of stapler used	60.0
Length of time spent on the waiting list for surgery	33.3

*Items where less than 70% of surgeons scored the item 7-9

Table 8: Core set 3b - Potential complications and side-effects of surgery (measured *during* or *after* surgery)

Item (n=7)*	Percentage scoring 7-9		
	Surgeons (n=68)	Other HCPs (n=55)	All (n=133)
Problems with bowel movements/flatulence**	61.5	68.6	64.7
Problems with teeth**	51.6	60.0	55.3
Hair loss	53.8	56.0	54.8
Problems with kidney stones**	43.1	56.0	48.7
Skin problems or irritations, e.g., rashes, sores, loose skin or ulcers or exacerbation of existing skin problems**	30.8	60.8	44.0
Problems with immune system, e.g., recurrent infections	32.3	46.0	38.3
Leg cramps	24.6	26.0	25.2

*Items where less than 70% of ALL participants combined scored the item 7-9

**Items the international patient advisory group felt were important to save

8. Which items are critically important for the core sets?

All items that have reached the stage of being included in a consensus meeting are generally agreed to be very important, but a core set should include only the critically important items. **We need your help to decide which items are critically important to include.**

Looking through the tables in section 6 you might have felt that some of the items overlap or link to others quite naturally. At the meeting we're going to decide whether some items should be grouped (collapsed) together – if you have a chance beforehand, please think about which items you might group together.
