# Supplementary material for: Development of a Bariatric Surgery Core Data Set for an International Registry
Source: Obes Surg. 2023 Mar 24;33(5):1463–75. doi: 10.1007/s11695-023-06545-y (PMC10156789; doi:10.1007/s11695-023-06545-y)
Supplement: Supplementary file 3 — Supplementary file3 (PDF 816 KB) [file 11695_2023_6545_MOESM3_ESM.pdf]

Questionnaire – Round 1

A1	Unique citation ID	

Development of CORE SETS for registries to monitor and evaluate bariatric surgery

Welcome to the International Bariatric Surgery Registry project

The International Federation for the Surgery of Obesity and Metabolic Disorders (IFSO) has set up a global registry project to allow for monitoring and comparisons of obesity and bariatric surgery on an international level, with the aim of improving patient care. Agreed standardised sets of information, also known as '**core sets'**, will optimise comparisons. Core sets include the information that should be **measured and reported *as a minimum* in all evaluations of a particular intervention**.

We are proposing 3 core sets, each representing a different time point at which information is collected in the registry.
Core set 1 (baseline information) will focus on information that is collected *only once before* surgery (baseline)
Core set 2 (effectiveness outcomes) will focus on information used to measure the impact or effectiveness of bariatric surgery - measured *both before and after* surgery
Core set 3 (surgical information) will focus on information measured *during or after* surgery, including information about the surgical procedure **(Set 3a - surgeons only)** and potential complications and side-effects of surgery **(Set 3b)**

Consensus among expert stakeholders (i.e. you) working in the field of bariatric surgery on the minimum core information to measure in each set in the registry is now needed. This is the first of two surveys where we will ask you to rate which information (data items) you think are **critical** to be measured and reported in a bariatric surgery registry as part of the core sets. The survey items are written in plain language, followed by medical terminology in brackets (where relevant).

Please rate each item using a Likert scale of 1-9. A score of **7-9 indicates an item that is of critical importance, 4-6 an item that is important but not critical and 1-3 an item of limited importance**. The scores will be analysed and used to develop a second survey, where you will see opinions of your peer group and the other stakeholder groups. At the end of the second survey, we plan to hold a consensus meeting where the final core sets will be agreed.

PLEASE NOTE: when rating items, please remember that the core sets represent the *minimum* that should be included in all bariatric registries internationally to allow comparisons. Development of the core sets will not preclude other information being collected in the registry.

	**First, a few things about you**	**Options**
A2	Professional specialty (please tick all that apply)	1, Bariatric Surgeon | 2, Bariatric Physician | 3, Specialist nurse | 4, Dietitian | 5, Psychologist | 6, Other
A3	Other speciality (please specify)	
A4	How many years have you worked in the bariatric surgery field?	1, < 5 | 2, 5-10 | 3, >10
A5	With which gender do you identify?	1, Male | 2, Female | 3, Other | 4, prefer not to say
A6	What is your age?	1, < 30 | 2, 30-39 | 3, 40-49 | 4, 50-59 | 5, 60-69 | 6, 70+ | 7, Prefer not to say
A7	In what country do you live?	Drop down list of all 195 countries

A8	So that we can send you the second survey, please provide your email address in the box below. **All data you provide will be kept anonymous.**	Mandatory
	**Core Set 1: Baseline only information**<br><br>This includes items that are measured *only once before* surgery (at baseline).<br><br>Please rate how **critical** you think it is that the following items are measured before surgery and recorded in a bariatric surgery registry. Select the number that best represents your opinion. **Reminder:** 7-9 'critical importance', 4-6 'important but not critical', 1-3 'limited importance'. If you feel unable to rate any of the items, please tick 'unable to answer'.	
	**i. Administrative information**	
1	Source of funding to pay for the surgery, e.g., private health care insurance, public funding/national health service, self-paying	**1 2 3 4 5 6 7 8 9**<br>**Unable to answer**
2	Date on which the patient was referred for surgery	
3	Details of which members of the multi-disciplinary team have been involved with the patient to date	
	**ii. Patient demographics**	
4	Sex of the patient	
5	Age of the patient	
6	Ethnicity of the patient	
7	Educational level of the patient	
8	Ability of patient to purchase/afford supplements for life, post-surgery	
	**iii. Clinical history**	
9	Height of the patient	
10	History of any previous bariatric surgery	
11	Details of previous weight loss programs	
12	Details of pre-surgery weight loss	
13	Time period over which pre-surgery weight loss occurred	
14	Duration of type 2 diabetes	
15	Other medical conditions not directly related to obesity e.g., type 1 diabetes, organ transplantation, dementia	

16	For this core set (Set 1 - Baseline only information), if there is anything else you think is ***critical*** to include in a bariatric surgery registry, please specify	
	**Core Set 2: Effectiveness outcomes**<br><br>This includes items that may be measured *both before and after surgery* to measure the impact or effectiveness of bariatric surgery.<br><br>Please rate how **critical** you think it is that the following items are measured in bariatric surgical registries. Select the number that best represents your opinion. **Reminder:** 7-9 critical importance, 4-6 important but not critical, 1-3 limited importance. If you feel unable to rate any of the items, please tick 'unable to answer'.<br><br>For each item, **please also indicate all timepoints you consider are critical to measure** the item (tick **all** that are critical)	
	**i. Obesity-related disease**	
17	Abnormal or irregular heartbeat, or use of medication (arrhythmia)	**1 2 3 4 5 6 7 8 9**<br>**Unable to answer**
	In addition to baseline, which time point(s) **after** surgery is the most important to measure this item? (tick all that apply)	6 weeks, 1 year, 2 years, 5 years, 5-10 years, more than 10 years, other
	Other (please specify)	
18	High blood pressure, or use of blood pressure medication (hypertension)	
19	Risk of future heart and vascular problems (assessment of cardiovascular risk)	
20	Congestive heart failure, or use of medication	
21	Diagnosis of Type 2 diabetes	
22	Medication for Type 2 diabetes	
23	How well the pancreas produces insulin (ß-cell function)	
24	Elevated fat and cholesterol in the blood, or use of medication (dyslipidemia)	
25	Problems with breathing during sleep (obstructive sleep apnoea)	
26	Ability to fall asleep at night or quality of sleep (sleep disorders other than sleep apnoea)	
27	Joint disease, or use of medication, or being considered for joint replacement	
28	Long standing acid reflux, or use of medication (gastro-esophageal reflux or GERD)	
29	Bladder problems (urinary incontinence)	
30	Long standing diseases of the lungs such as asthma (chronic pulmonary disease)	
31	Thyroid function, or use of medication (hypothyroidism)	

32	Obesity-related liver disease, e.g., non-alcoholic fatty liver disease	
33	Male or female reproductive function, e.g., polycystic ovary syndrome, infertility (reproductive dysfunction)	
34	Long standing fluid retention (lymphedema)	
35	Abnormal accumulation of fat in legs/arms (lipedema)	
	**ii. Mental health status assessed by a health professional** *(Note: patient-reported mental health will be measured separately)*	
36	Suicidal thoughts	
37	Binge eating	
38	Depression, or use of medication	
39	Feelings towards one's body shape or appearance (body dysmorphia/dysmorphic disorder)	
40	Addictive behaviours, e.g., alcohol, gambling, illicit drugs	
41	Anger management problems	
	**iii. Anthropometric (body measurement) data**	
42	Weight	
43	Body shape, e.g., waist and hip measurements	
	**iv. Lifestyle data**	
44	Alcohol	
45	Smoking	
46	Employment	
47	Changes in family and relationship	
	**v. Other outcomes**	
48	Changes in gut microbiota (gut flora)	
49	Use of weight loss medication	
50	For this core set (Set 2 - Effectiveness outcomes), if there is anything else you think is ***critical*** to include in a bariatric surgery registry, please specify	

	Set 3a: Surgical procedure information (surgeons only)<br><br>This section lists information related specifically to the surgical procedure. These items are measured ***only once*** around the time of surgery.<br><br>Please rate how **critical** you think it is that the following items are measured in bariatric surgical registries. Select the number that best represents your opinion.<br>**Reminder:** 7-9 critical importance, 4-6 important but not critical, 1-3 limited importance. If you feel unable to rate any of the items, please tick 'unable to answer'.	
	**i. General surgical information**	
51	Pre-operative assessment of surgical risk, e.g., OS-MRS score or similar	**1 2 3 4 5 6 7 8 9**<br>**Unable to answer**
52	Length of time spent on the waiting list for surgery	
53	Length of time spent in hospital after admission for surgery	
54	Name of surgical procedure, e.g., sleeve gastrectomy, one-anastomosis gastric bypass	
55	Surgical approach to gain access, e.g., laparoscopic, open or endoscopic	
	**ii. Stapling/suturing procedures**	
56	Height of staples used	
57	Make of stapler used	
58	Type of reinforcement used	
59	Size of bougie	
60	Distance between resection and pylorus (for sleeve gastrectomy only)	
61	Hiatus hernia repair undertaken	
62	Closure of hernia defects undertaken (not for sleeve gastrectomy)	
63	Measurements of limb length (not for sleeve gastrectomy)	
	**iii. Device procedures**	
64	Type/make of device (including band and balloon, adjustable or non-adjustable)	
65	Method of balloon placement, e.g., swallowed or endoscopically placed	
66	Fill volume of balloon	
67	Duration of balloon implantation (when removed)	
68	For this core set (Set 3a – Procedural information), if there is anything else you think is ***critical*** to include in a bariatric surgery registry, please specify	

	**Set 3b: Potential complications and side-effects of surgery**<br><br>The following section lists events that may occur *during or after* bariatric surgery. Please note, these are only possibilities and do not occur in everyone.<br><br>Please rate how **critical** you think it is that the following items are measured in bariatric surgical registries. Select the number that best represents your opinion. **Reminder**: 7-9 critical importance, 4-6 important but not critical, 1-3 limited importance. If you feel unable to rate any of the items, please tick 'unable to answer'.<br><br>For each item (where applicable), **please also indicate the timepoints you consider are critical to measure** this variable (tick **all** that are critical)

	**i. Death**	
69	Death from surgical complications whilst still in hospital (in-hospital mortality)	**1 2 3 4 5 6 7 8 9 Unable to answer**
	In addition to baseline, which time point(s) **after** surgery is the most important, relevant to this item? (tick all that apply)	6 weeks, 1 year, 2 years, 5 years, 5-10 years, more than 10 years, other
	Other (please specify)	
70	Death after discharge from hospital (post-discharge mortality)	
71	Cause of death	
	**ii. Technical complications of stapling/suturing procedures**	
72	Problems with anastomotic/staple line/suture line including subsequent infections	
73	Obstruction including ileus and/or hernia	
	**iii. Technical complications related to operations using devices**	
74	Complications that may occur shortly after the operation, when the patient is still in hospital	
75	Complications that occur sometime after the operation, once the patient has been discharged	
	**iv. General complications of surgery (early post-operative period)**	
76	Accidental damage to other organs during surgery (organ injury)	
77	Bleeding inside the body (intra-abdominal or endoluminal)	
78	Problems with the heart, vessels, or blood clots (cardiovascular problems or venous thromboembolism)	
79	Problems with the kidneys, including rhabdomyolysis (renal problems)	
80	Problems with gastric and/or stomal ulcers	
81	Unplanned use of high dependency, intensive care or critical care units	

82	Liver problems	
83	Feeling sick or vomiting (nausea)	
84	Whether a re-intervention occurred, including a classification of its severity, e.g., Clavien-Dindo or similar	
	**v. Side effects of surgery (longer-term)**	
85	Pain or discomfort in the body	
86	Problems with bowel movements/flatulence	
87	Problems swallowing or bringing food back up (dysphagia/regurgitation)	
88	Skin problems or irritations, e.g., rashes, sores, loose skin or ulcers or exacerbation of existing skin problems	
89	When food moves too quickly from the stomach into the small intestine causing symptoms such as cramps, diarrhea, nausea, feeling hot and sweaty (dumping syndrome)	
90	Problems with gallstones	
91	Problems with drops in blood sugar after a meal (reactive hypoglycaemia)	
92	Problems in bone strength (bone density)	
93	Problems with teeth	
94	Hair loss	
95	Problems with kidney stones	
96	Leg cramps	
97	Problems with immune system, e.g., recurrent infections	
	**vi. Nutritional outcomes**	
98	The amount and type of food patients consume (nutritional intake)	
99	Vitamin and mineral levels	
100	Clinical malnutrition	
101	For this core set (Set 3b – Potential complications and side effects of surgery), if there is anything else you think is ***critical*** to include in a bariatric surgery registry, please specify	
	**You have now finished the survey. Thank you for your time and support with this study. We will be in contact in a few weeks with the second survey.**	

Questionnaire – Round 2 (A) – Basic Feedback

A1	Unique citation ID				
	**Development of CORE SETS for registries to monitor and evaluate bariatric surgery – Round 2**<br><br>Welcome to the International Bariatric Surgery Registry project<br><br>Thank you for taking part of Round 1 of the survey earlier this year. This is the final of two surveys where we will ask you to rate which information (data items) you think are **critical** to be measured and reported in a bariatric surgery registry as part of core sets. If you'd like a reminder of the study information, please click here [insert link]<br><br>*[Information from Round 1 to be included in link:]*<br>[The International Federation for the Surgery of Obesity and Metabolic Disorders (IFSO) has set up a global registry project to allow for monitoring and comparisons of obesity and bariatric surgery on an international level, with the aim of improving patient care. Agreed standardised sets of information, also known as **'core sets'**, will optimise comparisons. Core sets include the information that should be **measured and reported *as a minimum* in all evaluations of a particular intervention**.<br><br>We are proposing 3 core sets, each representing a different time point at which information is collected in the registry.<br>**Core set 1 (baseline information)** will focus on information that is collected <u>*only once before*</u> surgery (baseline)<br>**Core set 2 (effectiveness outcomes)** will focus on information used to measure the impact or effectiveness of bariatric surgery - measured <u>*both*</u> <u>*before and after*</u> surgery<br>**Core set 3 (surgical information)** will focus on information measured <u>*during or after*</u> surgery, including information about the surgical procedure **(Set 3a - surgeons only)** and potential complications and side-effects of surgery **(Set 3b)**<br><br>Consensus among expert stakeholders (i.e. you) working in the field of bariatric surgery on the minimum core information to measure in each set in the registry is now needed.]<br><br>The survey items are written in plain language, followed by medical terminology in brackets (where relevant).<br><br>In this round, we have included your scores from Round 1 as well as the scores of your peer group and other health professional groups.<br>Please re-rate each item, considering the scores from Round 1. As a reminder, each item is rated on a Likert scale of 1-9. A score of **7-9 indicates an item that is of critical importance, 4-6 an item that is important but not critical and 1-3 an item of limited importance**.<br><br>**PLEASE NOTE: when rating items, please remember that the core sets represent the *minimum* that should be included in all bariatric registries internationally to allow comparisons. Development of the core sets will not preclude other information being collected in the registry**.				
	**Core Set 1: Baseline only information**<br><br>This includes items that are measured <u>*only once before*</u> surgery (at baseline).<br><br>Please rate how **critical** you think it is that the following items are measured before surgery and recorded in a bariatric surgery registry. Select the number that best represents your opinion. **Reminder:** 7-9 'critical importance', 4-6 'important but not critical', 1-3 'limited importance'. If you feel unable to rate any of the items, please tick 'unable to answer'. Round 1 scores are provided for your information.				
	**i. Administrative information**	**Your Round 1 score**	**[Professional group] Round 1 score**	**All other professions**	**1 2 3 4 5 6 7 8 9 Unable to answer**

				Round 1 score	
1	Source of funding to pay for the surgery, e.g., private health care insurance, public funding/national health service, self-paying				
2	Date on which the patient was referred for surgery				
3	Details of which members of the multi-disciplinary team have been involved with the patient to date				
	**ii. Patient demographics**				
4	Sex of the patient				
5	Age of the patient				
6	Ethnicity of the patient				
7	Educational level of the patient				
8	Ability of patient to purchase/afford supplements for life, post-surgery				
	**iii. Clinical history**				
9	Height of the patient				
10	History of any previous bariatric surgery				
11	History of any previous abdominal surgery (other than bariatric surgery)				
12	Weight history				
13	Details of previous weight loss programs				
14	Time period over which pre-surgery weight loss occurred				
15	Duration of type 2 diabetes				
16	Other medical conditions not directly related to obesity e.g., type 1 diabetes, organ transplantation, dementia				
17	Medication history				

	**Core Set 2: Effectiveness outcomes**<br><br>This includes items that may be measured *both before and after surgery* to measure the impact or effectiveness of bariatric surgery.<br><br>Please rate how **critical** you think it is that the following items are measured in bariatric surgical registries. Select the number that best represents your opinion. **Reminder:** 7-9 critical importance, 4-6 important but not critical, 1-3 limited importance. If you feel unable to rate any of the items, please tick 'unable to answer'. Round 1 scores are provided for your information.				
	**i. Obesity-related disease**	**Your Round 1 score**	**[Professional group] Round 1 score**	**All other professions Round 1 score**	**1 2 3 4 5 6 7 8 9 Unable to answer**
18	Abnormal or irregular heartbeat, or use of medication (arrhythmia)				
19	High blood pressure, or use of blood pressure medication (hypertension)				
20	Risk of future heart and vascular problems (assessment of cardiovascular risk)				
21	Congestive heart failure, or use of medication				
22	Diagnosis of Type 2 diabetes				
23	Medication for Type 2 diabetes				
24	How well the pancreas produces insulin (ß-cell function)				
25	Elevated fat and cholesterol in the blood, or use of medication (dyslipidemia)				
26	Problems with breathing during sleep (obstructive sleep apnoea)				
27	Ability to fall asleep at night or quality of sleep (sleep disorders other than sleep apnoea)				
28	Joint disease, or use of medication, or being considered for joint replacement				
29	Long standing acid reflux, or use of medication (gastro-esophageal reflux or GERD)				
30	Bladder problems (urinary incontinence)				
31	Long standing diseases of the lungs such as asthma (chronic pulmonary disease)				
32	Thyroid function, or use of medication (hypothyroidism)				
33	Obesity-related liver disease, e.g., non-alcoholic fatty liver disease				
34	Male or female reproductive function, e.g., polycystic ovary syndrome, infertility (reproductive dysfunction)				
35	Long standing fluid retention (lymphedema)				

36	Abnormal accumulation of fat in legs/arms (lipedema)	
	**ii. Mental health status assessed by a health professional** *(Note: patient-reported mental health will be measured separately)*	
37	Suicidal thoughts	
38	Binge eating	
39	Depression, or use of medication	
40	Feelings towards one's body shape or appearance (body dysmorphia/dysmorphic disorder)	
41	Addictive behaviours, e.g., alcohol, gambling, illicit drugs	
42	Anger management problems	
	**iii. Anthropometric (body measurement) data**	
43	Weight	
44	Body shape, e.g., waist and hip measurements	
	**iv. Lifestyle data**	
45	Alcohol	
46	Smoking	
47	Employment	
48	Changes in family and relationship	
49	Physical activity levels	
	**v. Other outcomes**	
50	Changes in gut microbiota (gut flora)	
51	Use of weight loss medication	
	**Set 3a: Surgical procedure information (surgeons only)**<br><br>This section lists information related specifically to the surgical procedure. These items are measured *only once* around the time of surgery.<br><br>Please rate how **critical** you think it is that the following items are measured in bariatric surgical registries. Select the number that best represents your opinion. **Reminder:** 7-9 critical importance, 4-6 important but not critical, 1-3 limited importance. If you feel unable to rate any of the items, please tick 'unable to answer'. Round 1 scores are provided for your information.	

	i. General surgical information	Your Round 1 score	Surgeons group Round 1 score	1 2 3 4 5 6 7 8 9 Unable to answer
52	Pre-operative assessment of surgical risk, e.g., OS-MRS score or similar			
53	Length of time spent on the waiting list for surgery			
54	Length of time spent in hospital after admission for surgery			
55	Name of surgical procedure, e.g., sleeve gastrectomy, one-anastomosis gastric bypass			
56	Surgical approach to gain access, e.g., laparoscopic, open or endoscopic			
	**ii. Stapling/suturing procedures**			
57	Height of staples used			
58	Make of stapler used			
59	Type of reinforcement used			
60	Size of bougie			
61	Distance between resection and pylorus (for sleeve gastrectomy only)			
62	Hiatus hernia repair undertaken			
63	Closure of hernia defects undertaken (not for sleeve gastrectomy)			
64	Measurements of limb length (not for sleeve gastrectomy)			
	**iii. Device procedures**			
65	Type/make of device (including band and balloon, adjustable or non-adjustable)			
66	Method of balloon placement, e.g., swallowed or endoscopically placed			
67	Fill volume of balloon			
68	Duration of balloon implantation (when removed)			

Set 3b: Potential complications and side-effects of surgery

The following section lists events that may occur *during or after* bariatric surgery. Please note, these are only possibilities and do not occur in everyone.

Please rate how **critical** you think it is that the following items are measured in bariatric surgical registries. Select the number that best represents your opinion. **Reminder**: 7-9 critical importance, 4-6 important but not critical, 1-3 limited importance. If you feel unable to rate any of the items, please tick 'unable to answer'. Round 1 scores are provided for your information.

	**i. Death**	**Your Round 1 score**	**[Professional group] Round 1 score**	**All other professions Round 1 score**	**1 2 3 4 5 6 7 8 9 Unable to answer**
69	Death from surgical complications whilst still in hospital (in-hospital mortality)				
70	Death after discharge from hospital (post-discharge mortality)				
71	Cause of death				
	**ii. Technical complications of stapling/suturing procedures**				
72	Problems with anastomotic/staple line/suture line including subsequent infections				
73	Obstruction including ileus and/or hernia				
	**iii. Technical complications related to operations using devices**				
74	Complications that may occur shortly after the operation, when the patient is still in hospital				
75	Complications that occur sometime after the operation, once the patient has been discharged				
	**iv. General complications of surgery (early post-operative period)**				
76	Accidental damage to other organs during surgery (organ injury)				
77	Bleeding inside the body (intra-abdominal or endoluminal)				
78	Problems with the heart, vessels, or blood clots (cardiovascular problems or venous thromboembolism)				
79	Problems with the kidneys, including rhabdomyolysis (renal problems)				
80	Problems with gastric and/or stomal ulcers				
81	Unplanned use of high dependency, intensive care or critical care units				
82	Liver problems				

83	Feeling sick or vomiting (nausea)	
84	Whether a re-intervention occurred, including a classification of its severity, e.g., Clavien-Dindo or similar	
	**v. Side effects of surgery (longer-term)**	
85	Pain or discomfort in the body	
86	Problems with bowel movements/flatulence	
87	Problems swallowing or bringing food back up (dysphagia/regurgitation)	
88	Skin problems or irritations, e.g., rashes, sores, loose skin or ulcers or exacerbation of existing skin problems	
89	When food moves too quickly from the stomach into the small intestine causing symptoms such as cramps, diarrhea, nausea, feeling hot and sweaty (dumping syndrome)	
90	Problems with gallstones	
91	Problems with drops in blood sugar after a meal (reactive hypoglycaemia)	
92	Problems in bone strength (bone density)	
93	Problems with teeth	
94	Hair loss	
95	Problems with kidney stones	
96	Leg cramps	
97	Problems with immune system, e.g., recurrent infections	
	**vi. Nutritional outcomes**	
98	The amount and type of food patients consume (nutritional intake)	
99	Vitamin and mineral levels	
100	Clinical malnutrition	
101	We will be inviting a sample of multidisciplinary bariatric health professionals to take part in an **online** consensus meeting on [date] to finalise the core outcome set. If you are interested in being invited to this, please tick this box. Ticking the box does **not** commit you to taking part in the meeting. Should you receive an invitation to the consensus meeting, you are free to decline participation.	

	**You have now finished the survey. Thank you for your time and support with this study.**	

Questionnaire – Round 2 (B) – Extended feedback

A1	Unique citation ID	

Development of CORE SETS for registries to monitor and evaluate bariatric surgery – Round 2

Welcome to the International Bariatric Surgery Registry project

Thank you for taking part of Round 1 of the survey earlier this year. This is the final of two surveys where we will ask you to rate which information (data items) you think are **critical** to be measured and reported in a bariatric surgery registry as part of core sets. If you'd like a reminder of the study information, please click here [insert link].

[Information from Round 1 to be included in link:]

[The International Federation for the Surgery of Obesity and Metabolic Disorders (IFSO) has set up a global registry project to allow for monitoring and comparisons of obesity and bariatric surgery on an international level, with the aim of improving patient care. Agreed standardised sets of information, also known as '**core sets'**, will optimise comparisons. Core sets include the information that should be **measured and reported *as a minimum* in all evaluations of a particular intervention**.

We are proposing 3 core sets, each representing a different time point at which information is collected in the registry.
Core set 1 (baseline information) will focus on information that is collected *only once before* surgery (baseline)
Core set 2 (effectiveness outcomes) will focus on information used to measure the impact or effectiveness of bariatric surgery - measured *both before and after* surgery
Core set 3 (surgical information) will focus on information measured *during or after* surgery, including information about the surgical procedure **(Set 3a - surgeons only)** and potential complications and side-effects of surgery **(Set 3b)**

Consensus among expert stakeholders (i.e. you) working in the field of bariatric surgery on the minimum core information to measure in each set in the registry is now needed.]

The survey items are written in plain language, followed by medical terminology in brackets (where relevant).

In this round, we have included the top 5 rated items from Round 1 for each of the survey sections. We have also included your scores from Round 1 as well as the scores of your peer group and other health professional groups. Please re-rate each item, considering the results and scores from Round 1. As a reminder, each item is rated on a Likert scale of 1-9. A score of **7-9 indicates an item that is of critical importance, 4-6 an item that is important but not critical and 1-3 an item of limited importance**.

PLEASE NOTE: when rating items, please remember that the core sets represent the *minimum* that should be included in all bariatric registries internationally to allow comparisons. Development of the core sets will not preclude other information being collected in the registry.

Core Set 1: Baseline only information

This includes items that are measured *only once before* surgery (at baseline).

Top five items from Round 1:

1. [insert item]
2. [insert item]
3. [insert item]
4. [insert item]

5. [insert item]

Please rate how **critical** you think it is that the following items are measured before surgery and recorded in a bariatric surgery registry. Select the number that best represents your opinion. **Reminder:** 7-9 'critical importance', 4-6 'important but not critical', 1-3 'limited importance'. If you feel unable to rate any of the items, please tick 'unable to answer'. Round 1 scores are provided for your information.

	**i. Administrative information**	**Your Round 1 score**	**[Professional group] Round 1 score**	**All other professions Round 1 score**	**1 2 3 4 5 6 7 8 9 Unable to answer**
1	Source of funding to pay for the surgery, e.g., private health care insurance, public funding/national health service, self-paying				
2	Date on which the patient was referred for surgery				
3	Details of which members of the multi-disciplinary team have been involved with the patient to date				
	**ii. Patient demographics**				
4	Sex of the patient				
5	Age of the patient				
6	Ethnicity of the patient				
7	Educational level of the patient				
8	Ability of patient to purchase/afford supplements for life, post-surgery				
	**iii. Clinical history**				
9	Height of the patient				
10	History of any previous bariatric surgery				
11	History of any previous abdominal surgery (other than bariatric surgery)				
12	Weight history				
13	Details of previous weight loss programs				
14	Time period over which any pre-surgery weight loss occurred				
15	Duration of type 2 diabetes				
16	Other medical conditions not directly related to obesity e.g., type 1 diabetes, organ transplantation, dementia				

17	Medication history	

Core Set 2: Effectiveness outcomes

This includes items that may be measured *both before and after surgery* to measure the impact or effectiveness of bariatric surgery.

Top five items from Round 1:

1. [insert item]
2. [insert item]
3. [insert item]
4. [insert item]
5. [insert item]

Please rate how **critical** you think it is that the following items are measured in bariatric surgical registries. Select the number that best represents your opinion. **Reminder:** 7-9 critical importance, 4-6 important but not critical, 1-3 limited importance. If you feel unable to rate any of the items, please tick 'unable to answer'. Round 1 scores are provided for your information.

	**i. Obesity-related disease**	**Your Round 1 score**	**[Professional group] Round 1 score**	**All other professions Round 1 score**	**1 2 3 4 5 6 7 8 9 Unable to answer**
18	Abnormal or irregular heartbeat, or use of medication (arrhythmia)				
19	High blood pressure, or use of blood pressure medication (hypertension)				
20	Risk of future heart and vascular problems (assessment of cardiovascular risk)				
21	Congestive heart failure, or use of medication				
22	Diagnosis of Type 2 diabetes				
23	Medication for Type 2 diabetes				
24	How well the pancreas produces insulin (ß-cell function)				
25	Elevated fat and cholesterol in the blood, or use of medication (dyslipidemia)				
26	Problems with breathing during sleep (obstructive sleep apnoea)				
27	Ability to fall asleep at night or quality of sleep (sleep disorders other than sleep apnoea)				
28	Joint disease, or use of medication, or being considered for joint replacement				
29	Long standing acid reflux, or use of medication (gastro-esophageal reflux or GERD)				
30	Bladder problems (urinary incontinence)				

31	Long standing diseases of the lungs such as asthma (chronic pulmonary disease)	
32	Thyroid function, or use of medication (hypothyroidism)	
33	Obesity-related liver disease, e.g., non-alcoholic fatty liver disease	
34	Male or female reproductive function, e.g., polycystic ovary syndrome, infertility (reproductive dysfunction)	
35	Long standing fluid retention (lymphedema)	
36	Abnormal accumulation of fat in legs/arms (lipedema)	
	**ii. Mental health status assessed by a health professional** *(Note: patient-reported mental health will be measured separately)*	
37	Suicidal thoughts	
38	Binge eating	
39	Depression, or use of medication	
40	Feelings towards one's body shape or appearance (body dysmorphia/dysmorphic disorder)	
41	Addictive behaviours, e.g., alcohol, gambling, illicit drugs	
42	Anger management problems	
	**iii. Anthropometric (body measurement) data**	
43	Weight	
44	Body shape, e.g., waist and hip measurements	
	**iv. Lifestyle data**	
45	Alcohol	
46	Smoking	
47	Employment	
48	Changes in family and relationship	
49	Physical activity levels	
	**v. Other outcomes**	
50	Changes in gut microbiota (gut flora)	
51	Use of weight loss medication	

	**Set 3a: Surgical procedure information (surgeons only)**<br><br>This section lists information related specifically to the surgical procedure. These items are measured *only once* around the time of surgery.<br><br>Top five items from Round 1:<br><br>1. [insert item]<br>2. [insert item]<br>3. [insert item]<br>4. [insert item]<br>5. [insert item]<br><br>Please rate how **critical** you think it is that the following items are measured in bariatric surgical registries. Select the number that best represents your opinion. **Reminder:** 7-9 critical importance, 4-6 important but not critical, 1-3 limited importance. If you feel unable to rate any of the items, please tick 'unable to answer'. Round 1 scores are provided for your information.			
	**i. General surgical information**	**Your Round 1 score**	**Surgeons group Round 1 score**	**1 2 3 4 5 6 7 8 9 Unable to answer**
52	Pre-operative assessment of surgical risk, e.g., OS-MRS score or similar			
53	Length of time spent on the waiting list for surgery			
54	Length of time spent in hospital after admission for surgery			
55	Name of surgical procedure, e.g., sleeve gastrectomy, one-anastomosis gastric bypass			
56	Surgical approach to gain access, e.g., laparoscopic, open or endoscopic			
	**ii. Stapling/suturing procedures**			
57	Height of staples used			
58	Make of stapler used			
59	Type of reinforcement used			
60	Size of bougie			
61	Distance between resection and pylorus (for sleeve gastrectomy only)			
62	Hiatus hernia repair undertaken			
63	Closure of hernia defects undertaken (not for sleeve gastrectomy)			

64	Measurements of limb length (not for sleeve gastrectomy)	
	**iii. Device procedures**	
65	Type/make of device (including band and balloon, adjustable or non-adjustable)	
66	Method of balloon placement, e.g., swallowed or endoscopically placed	
67	Fill volume of balloon	
68	Duration of balloon implantation (when removed)	

Set 3b: Potential complications and side-effects of surgery

The following section lists events that may occur ***during or after*** bariatric surgery. Please note, these are only possibilities and do not occur in everyone.

Top five items from Round 1:

1. [insert item]
2. [insert item]
3. [insert item]
4. [insert item]
5. [insert item]

Please rate how **critical** you think it is that the following items are measured in bariatric surgical registries. Select the number that best represents your opinion. **Reminder**: 7-9 critical importance, 4-6 important but not critical, 1-3 limited importance. If you feel unable to rate any of the items, please tick 'unable to answer'. Round 1 scores are provided for your information.

	**i. Death**	**Your Round 1 score**	**[Professional group] Round 1 score**	**All other professions Round 1 score**	**1 2 3 4 5 6 7 8 9 Unable to answer**
69	Death from surgical complications whilst still in hospital (in-hospital mortality)				
70	Death after discharge from hospital (post-discharge mortality)				
71	Cause of death				
	**ii. Technical complications of stapling/suturing procedures**				
72	Problems with anastomotic/staple line/suture line including subsequent infections				
73	Obstruction including ileus and/or hernia				
	**iii. Technical complications related to operations using devices**				

74	Complications that may occur shortly after the operation, when the patient is still in hospital	
75	Complications that occur sometime after the operation, once the patient has been discharged	
	**iv. General complications of surgery (early post-operative period)**	
76	Accidental damage to other organs during surgery (organ injury)	
77	Bleeding inside the body (intra-abdominal or endoluminal)	
78	Problems with the heart, vessels, or blood clots (cardiovascular problems or venous thromboembolism)	
79	Problems with the kidneys, including rhabdomyolysis (renal problems)	
80	Problems with gastric and/or stomal ulcers	
81	Unplanned use of high dependency, intensive care or critical care units	
82	Liver problems	
83	Feeling sick or vomiting (nausea)	
84	Whether a re-intervention occurred, including a classification of its severity, e.g., Clavien-Dindo or similar	
	**v. Side effects of surgery (longer-term)**	
85	Pain or discomfort in the body	
86	Problems with bowel movements/flatulence	
87	Problems swallowing or bringing food back up (dysphagia/regurgitation)	
88	Skin problems or irritations, e.g., rashes, sores, loose skin or ulcers or exacerbation of existing skin problems	
89	When food moves too quickly from the stomach into the small intestine causing symptoms such as cramps, diarrhea, nausea, feeling hot and sweaty (dumping syndrome)	
90	Problems with gallstones	
91	Problems with drops in blood sugar after a meal (reactive hypoglycaemia)	
92	Problems in bone strength (bone density)	
93	Problems with teeth	
94	Hair loss	
95	Problems with kidney stones	
96	Leg cramps	

97	Problems with immune system, e.g., recurrent infections	
	**vi. Nutritional outcomes**	
98	The amount and type of food patients consume (nutritional intake)	
99	Vitamin and mineral levels	
100	Clinical malnutrition	
101	We will be inviting a sample of multidisciplinary bariatric health professionals to take part in an **online** consensus meeting on [date] to finalise the core outcome set. If you are interested in being invited to this, please tick this box. Ticking the box does **not** commit you to taking part in the meeting. Should you receive an invitation to the consensus meeting, you are free to decline participation.<br><br>**You have now finished the survey. Thank you for your time and support with this study.**	
