# Supplementary material for: Development of a Bariatric Surgery Core Data Set for an International Registry
Source: Obes Surg. 2023 Mar 24;33(5):1463–75. doi: 10.1007/s11695-023-06545-y (PMC10156789; doi:10.1007/s11695-023-06545-y)
Supplement: Supplementary file 4 — Supplementary file4 (DOCX 38 KB) [file 11695_2023_6545_MOESM4_ESM.docx]

| **Questionnaire item** | | **Percentage of All participants scoring 7-9 in Delphi survey** | | **Consensus meeting action (see Table 1)** | **Consensus meeting outcome (***% of participants who voted ‘IN’***)** |
| --- | --- | --- | --- | --- | --- |
|  |  | **Round 1** | **Round 2** |  |  |
| **Core set 1: Baseline only information** | | | | | |
| 1. | Source of funding to pay for the surgery, e.g., private health care insurance, public funding/national health service, self-paying | 53.3 | 54.9 | *Ratify ‘OUT’* | *OUT* |
| 2. | Date on which the patient was referred for surgery | 44.5 | 35.5 | *Ratify ‘OUT’* | *OUT* |
| 3 | Details of which members of the multi-disciplinary team have been involved with the patient to date | 70.9 | 70.2 | *DISCUSS* | *OUT (no vote)* |
| 4 | Sex of the patient | 62.2 | 77.0 | *DISCUSS* | *IN (no vote as all groups put this forward)* |
| 5 | Age of the patient | 75.6 | 84.4 | *DISCUSS* | *IN (no vote as all groups put this forward)* |
| 6 | Ethnicity of the patient | 41.1 | 45.1 | *Ratify ‘OUT’* | *OUT* |
| 7 | Educational level of the patient | 40.7 | 39.3 | *Ratify ‘OUT’* | *OUT* |
| 8 | Ability of patient to purchase/afford supplements for life, post-surgery | 58.2 | 60.8 | *Ratify ‘OUT’* | *OUT* |
| 9 | Height of the patient | 75.4 | 88.5 | *DISCUSS* | *IN (no vote as all groups put this forward)* |
| 10 | History of any previous bariatric surgery | 96.1 | 95.9 | *Ratify ‘IN’* | *IN (75%)* – change wording to ‘any previous bariatric procedure’ |
| 11 | Details of previous weight loss programs | 65.0 | 62.8 | *Ratify ‘OUT’* | *OUT* |
| 12 | Details of pre-surgery weight loss (changed to Weight history) | 65.0 | 77.7 | *DISCUSS* | *OUT (no vote)* |
| 13 | Time period over which pre-surgery weight loss occurred | 53.1 | 71.1 | *DISCUSS* | *OUT (no vote)* |
| 14 | Duration of type 2 diabetes | 81.8 | 51.2 | *Ratify ‘OUT’* | *OUT* |
| 15 | Other medical conditions not directly related to obesity e.g., type 1 diabetes, organ transplantation, dementia | 77.7 | 87.7 | *DISCUSS* | *OUT* |
| 16 | History of any previous abdominal surgery (other than bariatric surgery) **added after Round 1** | N/A | 78.7 | *DISCUSS* | *OUT (no vote)* |
| 17 | Medication history **added after Round 1** | N/A | 84.4 | *DISCUSS* | *OUT (no vote)* |
| **Core set 2: Effectiveness outcomes** | | | | | |
| 18 | Abnormal or irregular heartbeat, or use of medication (arrhythmia) | 66.7 | 78.0 | *DISCUSS* | *OUT (no vote)* |
| 19 | High blood pressure, or use of blood pressure medication (hypertension) | 82.7 | 89.0 | *DISCUSS* | *OUT (57%)* |
| 20 | Risk of future heart and vascular problems (assessment of cardiovascular risk) | 69.6 | 80.3 | *DISCUSS* | *OUT (48%)* |
| 21 | Congestive heart failure, or use of medication | 78.2 | 87.2 | *DISCUSS* | *OUT (no vote)* |
| 22 | Diagnosis of Type 2 diabetes | 96.9 | 97.5 | *Ratify ‘IN’* | *IN (76%)* – combine with ‘medication for type 2 diabetes (Voted ‘Yes’ by 95%)’ |
| 23 | Medication for Type 2 diabetes | 93.2 | 94.1 | *DISCUSS* | *IN (90%)* – combine with ‘diagnosis of type 2 diabetes (Voted ‘Yes’ by 95%)’ |
| 24 | How well the pancreas produces insulin (ß-cell function) | 58.9 | 62.9 | *Ratify ‘OUT’* | *OUT* |
| 25 | Elevated fat and cholesterol in the blood, or use of medication (dyslipidaemia) | 77.0 | 83.1 | *DISCUSS* | *OUT (no vote)* |
| 26 | Problems with breathing during sleep (obstructive sleep apnoea) | 90.4 | 94.1 | *DISCUSS* | *OUT (no vote)* |
| 27 | Ability to fall asleep at night or quality of sleep (sleep disorders other than sleep apnoea) | 52.3 | 62.2 | *Ratify ‘OUT’* | *OUT* |
| 28 | Joint disease, or use of medication, or being considered for joint replacement | 69.2 | 74.6 | *DISCUSS* | *OUT (no vote)* |
| 29 | Long standing acid reflux, or use of medication (gastro-esophageal reflux or GERD) | 86.6 | 94.0 | *DISCUSS* | *IN (86%)* |
| 30 | Bladder problems (urinary incontinence) | 42.0 | 28.2 | *Ratify ‘OUT’* | *OUT* |
| 31 | Long standing diseases of the lungs such as asthma (chronic pulmonary disease) | 61.1 | 64.4 | *Ratify ‘OUT’* | *OUT* |
| 32 | Thyroid function, or use of medication (hypothyroidism) | 56.3 | 61.5 | *Ratify ‘OUT’* | *OUT* |
| 33 | Obesity-related liver disease, e.g., non-alcoholic fatty liver disease | 81.5 | 88.9 | *DISCUSS* | *OUT (33%)* |
| 34 | Male or female reproductive function, e.g., polycystic ovary syndrome, infertility (reproductive dysfunction) | 70.4 | 75.6 | *DISCUSS* | *OUT (no vote)* |
| 35 | Long standing fluid retention (lymphedema) | 46.6 | 41.0 | *Ratify ‘OUT’* | *OUT* |
| 36 | Abnormal accumulation of fat in legs/arms (lipedema) | 39.8 | 35.9 | *Ratify ‘OUT’* | *OUT* |
| 37 | Suicidal thoughts | 80.3 | 90.8 | *DISCUSS* | *OUT (no vote)* |
| 38 | Binge eating | 82.3 | 89.9 | *DISCUSS* | *OUT (no vote)* |
| 39 | Depression, or use of medication | 79.4 | 92.4 | *DISCUSS* | *OUT (33%)* |
| 40 | Feelings towards one's body shape or appearance (body dysmorphia/dysmorphic disorder) | 65.2 | 78.2 | *DISCUSS* | *OUT (no vote)* |
| 41 | Addictive behaviours, e.g., alcohol, gambling, illicit drugs | 79.8 | 94.1 | *DISCUSS* | *OUT (38%)* |
| 42 | Anger management problems | 43.9 | 38.7 | *Ratify ‘OUT’* | *OUT* |
| 43 | Weight | 94.6 | 96.6 | *Ratify ‘IN’* | *IN (100%)* |
| 44 | Body shape, e.g., waist and hip measurements | 61.6 | 71.2 | *DISCUSS* | *OUT (no vote)* |
| 45 | Alcohol | 79.8 | 91.6 | *DISCUSS* | *OUT (no vote)* |
| 46 | Smoking | 77.0 | 90.7 | *DISCUSS* | *OUT (no vote)* |
| 47 | Employment | 39.3 | 38.1 | *Ratify ‘OUT’* | *OUT* |
| 48 | Changes in family and relationship | 51.0 | 55.5 | *Ratify ‘OUT’* | *OUT* |
| 49 | Changes in gut microbiota (gut flora) | 37.1 | 33.9 | *Ratify ‘OUT’* | *OUT* |
| 50 | Use of weight loss medication | 68.6 | 75.2 | *DISCUSS* | *OUT (no vote)* |
| 51 | Physical activity levels **added after Round 1** | N/A | 78.2 | *DISCUSS* | *OUT (no vote)* |
| **Core set 3a: Surgical procedure information (surgeons only)** | | | | | |
| 52 | Pre-operative assessment of surgical risk, e.g., OS-MRS score or similar | 77.3 | 87.9 | *DISCUSS* | *OUT (24%)* |
| 53 | Length of time spent on the waiting list for surgery | 41.3 | 33.3 | *Ratify ‘OUT’* | *OUT* |
| 54 | Length of time spent in hospital after admission for surgery | 57.3 | 66.7 | *Ratify ‘OUT’* | *OUT* |
| 55 | Name of surgical procedure, e.g., sleeve gastrectomy, one-anastomosis gastric bypass | 95.5 | 97.0 | *Ratify ‘IN’* | *IN (100%)* |
| 56 | Surgical approach to gain access, e.g., laparoscopic, open or endoscopic | 93.6 | 95.5 | *Ratify ‘IN’* | *OUT (47%)* |
| 57 | Height of staples used | 63.6 | 74.2 | *DISCUSS* | *OUT (no vote)* |
| 58 | Make of stapler used | 53.2 | 60.0 | *Ratify ‘OUT’* | *OUT* |
| 59 | Type of reinforcement used | 58.7 | 73.8 | *DISCUSS* | *OUT (no vote)* |
| 60 | Size of bougie | 78.2 | 86.4 | *DISCUSS* | *OUT (no vote)* |
| 61 | Distance between resection and pylorus (for sleeve gastrectomy only) | 77.3 | 83.1 | *DISCUSS* | *OUT (no vote)* |
| 62 | Hiatus hernia repair undertaken | 86.4 | 90.9 | *DISCUSS* | *OUT (24%)* |
| 63 | Closure of hernia defects undertaken (not for sleeve gastrectomy) | 90.9 | 97.0 | *Ratify ‘IN’* | *OUT (38%)* |
| 64 | Measurements of limb length (not for sleeve gastrectomy) | 89.0 | 98.5 | *Ratify ‘IN’* | *OUT (50%)* and voted *OUT (65%)* again following discussion after breakout groups |
| 65 | Type/make of device (including band and balloon, adjustable or non-adjustable) | 76.9 | 87.3 | *DISCUSS* | *OUT (no vote)* |
| 66 | Method of balloon placement, e.g., swallowed or endoscopically placed | 69.7 | 83.6 | *DISCUSS* | *OUT (no vote)* |
| 67 | Fill volume of balloon | 71.7 | 88.5 | *DISCUSS* | *OUT (no vote)* |
| 68 | Duration of balloon implantation (when removed) | 79.8 | 91.9 | *DISCUSS* | *OUT (no vote)* |
| **Core set 3b: Potential complications and side-effects of surgery** | | | | | |
| 69 | Death from surgical complications whilst still in hospital (in-hospital mortality) | 96.3 | 96.6 | *Ratify ‘IN’* | *IN (82%)* |
| 70 | Death after discharge from hospital (post-discharge mortality) | 94.7 | 94.8 | *DISCUSS* | *OUT (no vote)* |
| 71 | Cause of death | 94.7 | 94.8 | *DISCUSS* | *OUT (48%)* |
| 72 | Problems with anastomotic/staple line/suture line including subsequent infections | 93.8 | 97.4 | *Ratify ‘IN’* | *OUT (55%)* |
| 73 | Obstruction including ileus and/or hernia | 95.5 | 96.5 | *Ratify ‘IN’* | *OUT (32%)* |
| 74 | Complications that may occur shortly after the operation when the patient is still in hospital | 95.5 | 96.5 | *Ratify ‘IN’* | *OUT (68%)* |
| 75 | Complications that occur sometime after the operation, once the patient has been discharged | 92.6 | 96.5 | *Ratify ‘IN’* | *OUT (64%)* |
| 76 | Accidental damage to other organs during surgery (organ injury) | 88.3 | 96.5 | *Ratify ‘IN’* | *OUT (18%)* |
| 77 | Bleeding inside the body (intra-abdominal or endoluminal) | 93.9 | 97.4 | *Ratify ‘IN’* | *OUT (45%)* |
| 78 | Problems with the heart, vessels, or blood clots (cardiovascular problems or venous thromboembolism) | 94.4 | 97.4 | *Ratify ‘IN’* | *OUT (50%)* |
| 79 | Problems with the kidneys, including rhabdomyolysis (renal problems) | 85.8 | 93.9 | *DISCUSS* | *OUT (no vote)* |
| 80 | Problems with gastric and/or stomal ulcers | 89.0 | 93.9 | *DISCUSS* | *OUT (no vote)* |
| 81 | Unplanned use of high dependency, intensive care or critical care units | 90.4 | 93.9 | *DISCUSS* | *OUT (17%)* |
| 82 | Liver problems | 83.7 | 91.2 | *DISCUSS* | *OUT (no vote)* |
| 83 | Feeling sick or vomiting (nausea) | 62.9 | 74.8 | *DISCUSS* | *OUT (no vote)* |
| 84 | Whether a re-intervention occurred, including a classification of its severity, e.g., Clavien-Dindo or similar | 93.5 | 98.2 | *Ratify ‘IN’* | *IN (82%)* |
| 85 | Pain or discomfort in the body | 62.7 | 77.6 | *DISCUSS* | *OUT (no vote)* |
| 86 | Problems with bowel movements/flatulence | 53.5 | 64.7 | *Ratify ‘OUT’* | *OUT* |
| 87 | Problems swallowing or bringing food back up (dysphagia/regurgitation) | 79.6 | 91.4 | *DISCUSS* | *OUT (no vote)* |
| 88 | Skin problems or irritations, e.g., rashes, sores, loose skin or ulcers or exacerbation of existing skin problems | 45.9 | 44.0 | *Ratify ‘OUT’* | *OUT* |
| 89 | When food moves too quickly from the stomach into the small intestine causing symptoms such as cramps, diarrhoea, nausea, feeling hot and sweaty (dumping syndrome) | 74.7 | 85.3 | *DISCUSS* | *OUT (no vote)* |
| 90 | Problems with gallstones | 70.3 | 81.0 | *DISCUSS* | *OUT (no vote)* |
| 91 | Problems with drops in blood sugar after a meal (reactive hypoglycaemia) | 77.7 | 87.8 | *DISCUSS* | *OUT (no vote)* |
| 92 | Problems in bone strength (bone density) | 64.1 | 73.9 | *DISCUSS* | *OUT (no vote)* |
| 93 | Problems with teeth | 49.7 | 55.3 | *Ratify ‘OUT’* | *OUT* |
| 94 | Hair loss | 50.5 | 54.8 | *Ratify ‘OUT’* | *OUT* |
| 95 | Problems with kidney stones | 47.7 | 48.7 | *Ratify ‘OUT’* | *OUT* |
| 96 | Leg cramps | 37.1 | 25.2 | *Ratify ‘OUT’* | *OUT* |
| 97 | Problems with immune system, e.g., recurrent infections | 45.6 | 38.3 | *Ratify ‘OUT’* | *OUT* |
| 98 | The amount and type of food patients consume (nutritional intake) | 74.1 | 87.9 | *DISCUSS* | *OUT (no vote)* |
| 99 | Vitamin and mineral levels | 88.9 | 96.6 | *Ratify ‘IN’* | *OUT (45%)* |
| 100 | Clinical malnutrition | 88.9 | 98.3 | *Ratify ‘IN’* | *OUT (64%)* but *IN* following discussion after breakout groups where all groups put it IN so no vote needed |
